# Fulfilling multiple roles in PROTAC design: The emerging potential of oligonucleotides

**DOI:** 10.1016/j.ejmech.2026.118688

**Published:** 2026-02-13

**Authors:** Daniel Alencar Rodrigues, Gustavo Salgado Pires, Urbi Roy, Eric Conway, Pedro de Sena Murteira Pinheiro

**Affiliations:** a School of Pharmacy and Biomolecular Sciences (PBS), Royal College of Surgeons in Ireland, 123 St Stephen’s Green, 2, Dublin, Ireland; b Laboratório de Avaliação e Síntese de Substâncias Bioativas (LASSBio), Instituto de Ciências Biomédicas, Universidade Federal do Rio de Janeiro, Cidade Universitária, Rio de Janeiro, 21941-902, Brazil; c Programa de Pós-Graduação em Farmacologia e Química Medicinal (PPGFQM), Instituto de Ciências Biomédicas, Universidade Federal do Rio de Janeiro, Cidade Universitária, Rio de Janeiro, 21941-902, Brazil; d School of Biomolecular and Biomedical Science, University College Dublin, 4, Dublin, Ireland

## Abstract

The field of targeted protein degradation (TPD) has emerged as a novel therapeutic approach based on event-driven pharmacology, rather than on continuous occupancy or inhibition of a target. Proteolysis-targeting chimeras (PROTACs) are one of the main TPD modalities, enabling a protein of interest (POI) to be brought into proximity with a ubiquitin ligase, which facilitates ubiquitination of the target protein, leading to its degradation. The design of PROTACs involves the development of heterobifunctional compounds composed of three parts: a POI ligand, a linker, and an E3 ligase ligand. To date, PROTAC technology has been applied to degrade a wide range of targets, with particular interest in previously “undruggable” targets, such as transcription factors. In this context, oligonucleotides have emerged as potential POI binders for transcription factors, since these targets naturally bind to DNA to modulate gene expression. This has led to the development of oligonucleotide-based degraders. Moreover, the role of oligonucleotides has expanded beyond their use as POI ligands. Several strategies have been explored to overcome inherent challenges such as poor stability and limited drug delivery. Currently, novel approaches also demonstrate the potential of nucleotides to function as linkers, where they can exert control over the spatial orientation between the POI ligand and the E3 ligase ligand, thereby optimizing ternary complex formation. In addition, nucleotides can also serve as E3 ligase ligands. Oligonucleotides represent a promising approach in PROTAC design, capable of fulfilling multiple functional roles.

## 1 Introduction

Conventional drug discovery relies on designing small molecules that bind to specific protein targets, aiming to modulate their activity and produce therapeutic effects. This occupancy-driven approach has proven effective for proteins with well-defined active or allosteric sites, such as enzymes and receptors. However, its limitations are evident when addressing targets lacking accessible binding pockets or presenting dynamic and complex structures [1–3]. A wide range of targets still pose a challenge in the development of drugs with high affinity and selectivity, being considered “undruggable” targets. Among these are proteins without enzymatic or signal transduction activity, such as scaffolding proteins and transcription factors (TFs), as well as those that depend on protein–protein interactions (PPIs), due to the unavailability of efficient ligand-binding pockets. The difficulty in achieving pico-to-nanomolar range potency in such cases may lead to high drug dose requirements, increasing the risk of *off*-target interactions and, consequently, a higher likelihood of adverse effects. In this sense, over the past 20 years, targeted protein degradation (TPD) has emerged as a novel therapeutic alternative based on an event-driven mode of action, that is, involving mechanisms in which a therapeutic effect is triggered by a specific cellular event, rather than by continuous occupancy or inhibition of a target. The TPD strategy takes advantage of endogenous protein degradation machinery, inducing the depletion or reduction of proteins of interest (POIs) [1–6].

In this context, proteolysis-targeting chimeras (PROTACs) have gained prominence as one of the main TPD strategies, being first developed in the early 2000s by Crews and co-workers as small molecules capable of bringing a POI into proximity with the ubiquitin-dependent proteasome system (UPS), facilitating the polyubiquitination of the POI and directing it for proteasomal degradation [7,8]. For this purpose, PROTACs are designed as bifunctional molecules containing a target-binding moiety, an E3 ubiquitin ligase recruiter, and a linker that connects these two subunits. A variety of E3 ligases are used as targets for the design of PROTACs. Although the human genome encodes more than 600 E3 ubiquitin ligases, the vast majority of PROTACs developed to date recruit only a few, with Cereblon (CRBN) and Von Hippel–Lindau (VHL) being the most widely used. This predominance is largely due to the availability of validated ligands with favorable properties — such as appropriate molecular weight and solubility — as well as the ubiquitous expression of these ligases in the human body [9,10]. This interaction favors the spatial approximation with UPS and the formation of a ternary complex POI-PROTAC-E3 that promotes the polyubiquitination [4–6,11]. Ubiquitination is a post-translational modification in which ubiquitin is covalently linked to lysine residues on the surface of a substrate protein via the ε-amino group. Subsequent to the POI degradation, PROTAC molecules are once again released in the cytoplasm, which makes them capable of targeting multiple POI units for degradation [12,13].

Undruggable targets account for 80-85% of disease-related targets present in the human proteome [14,15]. TFs are one of the greatest examples of undruggable targets that remain of great therapeutic interest due to their ability to specifically bind to DNA, recruit cofactor proteins and modulate target gene expression. Accordingly, TFs are involved in numerous biological processes, such as differentiation, immune responses, metabolism, and cell death. This interest rendered many attempts to inhibit the activity of transcription factors, highlighting oligonucleotide decoys as one of the most prominent options [16–18]. An oligonucleotide decoy is a synthetic short strand of DNA or RNA designed to mimic the physiological target sequence TFs or other DNA-binding proteins, allowing these ligands to bind to the target protein and prevent them from interacting with its endogenous target sequence, such as STAT3 [19]. Despite their therapeutic potential, oligonucleotides frequently exhibit limited potency, and the absence of efficient delivery strategies continues to hinder the development of new oligonucleotide-based therapeutics [20]. In this regard, PROTACs incorporating oligonucleotide decoys represents a promising strategy for expanding the range of targetable proteins for degradation with high potency and specificity. Moreover, this is particularly advantageous because DNA-binding sequences are often known, facilitating ligand design. It is important to notice, this subject has already been reviewed elsewhere [16,21,22]. However, the rapid growth and increasing complexity of research in this emerging field underscore the need for an updated and comprehensive overview, which this review aims to provide, specially due to the fact that the role of oligonucleotides have been expanded, being used not only as POI ligands, but as linkers and E3 ligase ligands as well.

## 2 Oligonucleotides as POI ligands in the design of PROTACs

### 2.1 Oligonucleotides decoys-based PROTACs

Oligonucleotides have emerged as a powerful alternative for guiding PROTAC-mediated degradation, particularly for proteins that naturally bind nucleic acids. By exploiting the inherent sequence specificity of DNA or RNA oligos, researchers have begun to design chimeric molecules that bridge POIs (TF binders) with E3 ligases. Integration of oligonucleotide decoys into PROTAC design has led to the development of innovative platforms, such as oligoTRAFTAC, O’PROTAC, and TF-PROTAC, which leverage nucleic acid–mediated binding and expand the scope of degradable targets beyond what was previously possible. These platforms were independently described by different research groups to induce the degradation of transcription factors using decoy oligonucleotides as POI recruiters. While they share similarities in design, they exploit different chemistries for linking the decoy oligonucleotide to the E3 ligase ligand. This section explores recent advances in oligonucleotide decoy–based PROTACs.

#### 2.1.1 TRAFTACs and oligoTRAFTACs platform

**Fig. 1:**
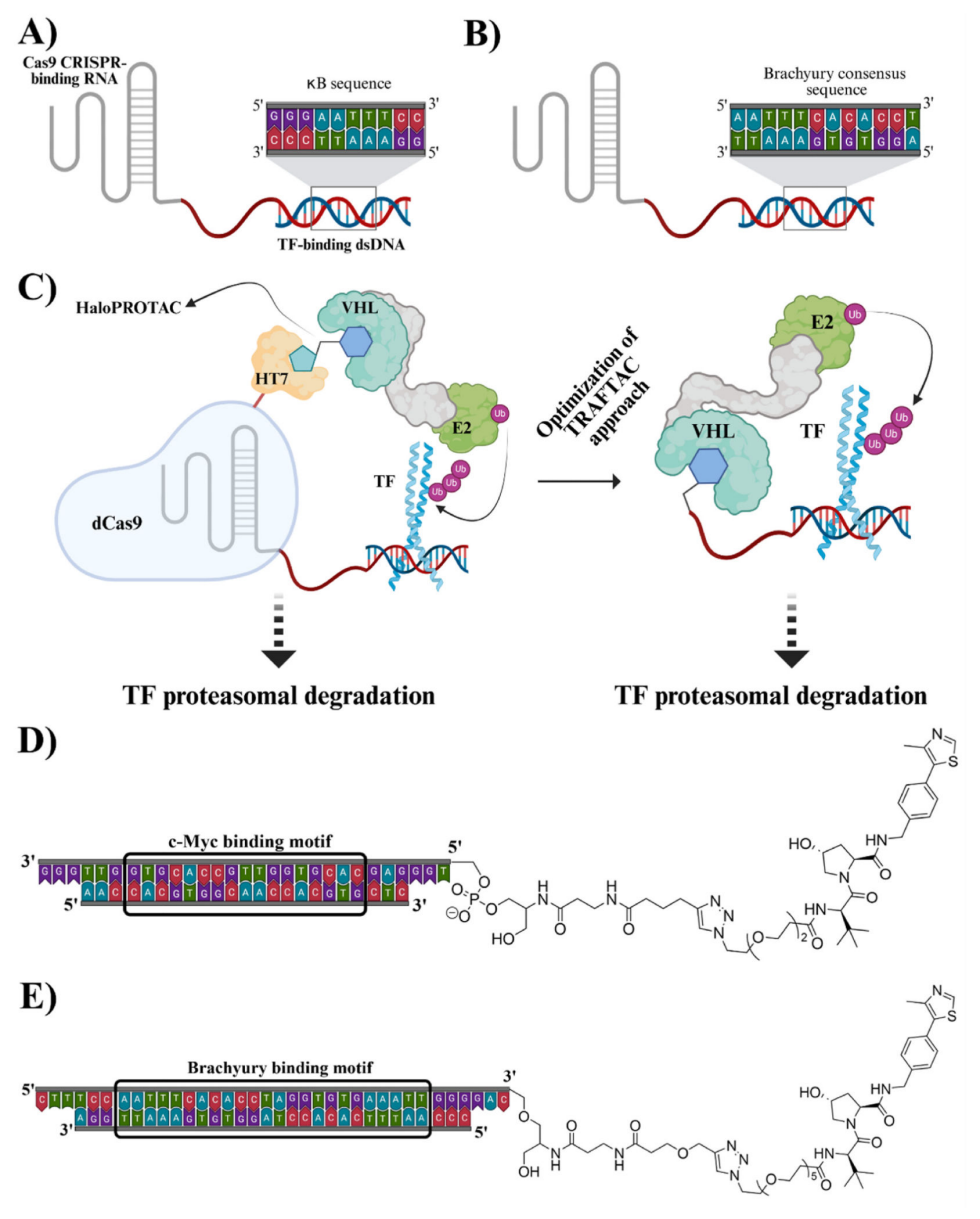
Targeted Degradation of Transcription Factors via TRAFTAC and OligoTRAFTAC approaches. **A**) The κB binding motif recognized by NF-κB is part of a chimeric nucleic acid-protein compound that uses CRISPR RNA (crRNA) to bind to catalytically dead dCas9 which is fused to the HaloTag7 protein tag. **B**) A similar strategy is shown for the brachyury consensus binding sequence using a different dsDNA. **C**) Mechanism of TRAFTAC approach. A bifunctional HaloPROTAC molecule bridges the HaloTag (HT7) domain fused to dCas9 and the E3 ligase VHL, facilitating ubiquitination and proteasomal degradation of the bound TF. OligoTRAFTACs do not require the ectopically expressed dCas9HT7 fusion protein for targeted degradation of TF. **D**) A chemically modified DNA oligonucleotide containing the c-Myc binding motif is conjugated to an E3 ligase ligand, allowing recruitment of an E3 ligase to target c-Myc for degradation. **E**) A similar construct with the brachyury binding motif enables targeted degradation of brachyury. Figure partially created with BioRender.com.

Samarasinghe and colleagues (2021) developed a novel approach for the targeted degradation of TF called TRAnscription Factor TArgeting Chimeras (TRAFTACs) [23]. This method involves heterobifunctional chimeric oligonucleotides with a transcription factor-specific DNA sequence attached to an E3 ligase-recruiting moiety. The approach operates by the chimeric oligo binding to its respective targets: the DNA sequence interacts to the transcription factor of interest (TOI), while the Cas9-binding crRNA section binds to an ectopically expressed fusion protein-dCas9 fused to HaloTag7 (dCas9HT7) (Fig. 1A and B). The addition of a HaloPROTAC molecule recruits the VHL-E3 ligase to the complex, bringing it into close proximity to the DNA-bound TOI. This results in ubiquitination and proteasomal degradation of the TOI (Fig. 1C). By using this approach, the authors successfully induced the degradation of NF-κB *in vitro* (Fig. 1A). To assess the broader applicability of their approach, they designed a TRAFTAC for the degradation of brachyury, a TF implicated in developmental biology and cancer (Fig. 1B). Remarkably, they demonstrated degradation of brachyury both *in vitro* and *in vivo*, thereby offering insights into TF degradation, surpassing the dynamics that can be achieved with small interfering RNA or CRISPR-mediated knockdown or knockout techniques [23]. In addition, TRAFTAC performance exhibited a clear structure-activity relationship (SAR) linked to E3 recruitment geometry and overall architecture: with the haloPROTAC, short linkers preferentially drove degradation of the CT-dCas9HT7 scaffold, whereas intermediate linkers enabled productive degradation of the TF target (p50/NF-κB and brachyury), and very long linkers lost target-degrading activity. Activity was also dependent on functional VHL engagement, as the inactive epimer failed to induce degradation, and on the presence of a cognate dsDNA sequence in the TRAFTAC (scrambled controls did not degrade), underscoring that TF recognition governs selectivity. Finally, HaloTag7 placement on dCas9 (N- vs C-terminal) shifted ubiquitination/degradation between scaffold and target, and for NF-κB, the requirement for TNF-α stimulation suggests degradation is favored when the TF is in a DNA-binding–competent (active) state. Despite its potential, the TRAFTAC system requires the co-delivery of both the chimeric oligo and the dCas9HT7 fusion protein, which poses challenges for bioavailability and therapeutic application. To address this limitation, second-generation TRAFTACs were developed in 2022, termed “oligoTRAFTACs”, this single-component approach uses only an oligonucleotide sequence linked to an E3 ligase-recruiting small molecule ligand, leading to the recruitment of the TOI and the cellular ubiquitination machinery (Fig. 1C). In this approach, the authors synthesized oligoTRAFTACs using the binding sequence of c-Myc (Fig. 1D) and brachyury (Fig. 1E) with a terminal alkyne group at either the 3′ or 5′ end and clicked through copper-catalyzed alkyne–azide cycloaddition (CuAAC) with azide-containing VHL ligand. OligoTRAFTACs degraded c-Myc and brachyury in HeLa and chordoma cell lines, respectively. Degradative activity was strongly influenced by where the VHL ligand was placed on the TF-binding oligonucleotide, the VHL-linker length, and the oligonucleotide backbone chemistry. For c-Myc, appending the VHL ligand at either the 5′ or 3′ end (OT7 vs OT10) produced comparable degradation, consistent with the use of flanking nucleotides that provided flexibility between the recruiter and the TF-binding element. In contrast, for brachyury, moving the VHL ligand to the 3′ end (OT3/OT4) improved degradation relative to 5′-modified constructs (OT1/OT2), and a shorter VHL-linker (2 PEG) was required for activity in the 5′-modified series (OT2 was active vs OT1 was inactive), indicating a geometry-sensitive ubiquitination window. Finally, switching from a phosphodiester to a phosphorothioate (PS) backbone (OT17) increased potency/stability—enabling endogenous brachyury degradation at lower nM in chordoma cells and *in vivo* activity in zebrafish—whereas the phosphodiester analog (OT3) did not produce the zebrafish phenotype, consistent with reduced nuclease resistance. *In vivo* experiments using zebrafish demonstrated the potential application of oligoTRAFTACs as a translational tool without the need for multiple components or genetic engineering [24].

#### 2.1.2 O’PROTACs platform

**Fig. 2:**
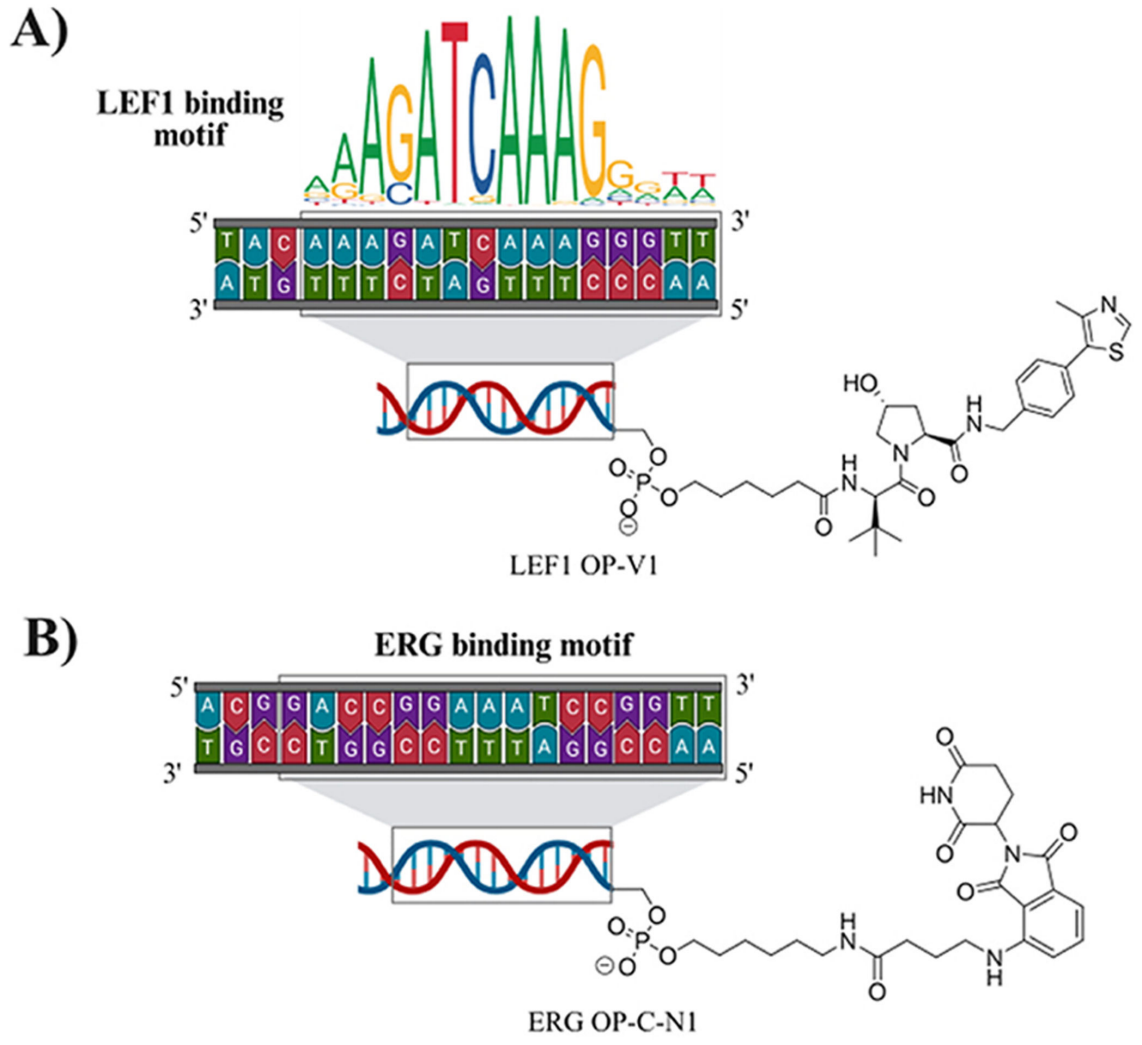
O’PROTACs for the degradation of LEF1 and ERG. **A**) LEF1 OP-V1 is an O’PROTAC containing a double-stranded oligonucleotide that mimics the LEF1 binding motif (AGATCAAAG), linked to VH032 for VHL recruitment. **B**) ERG OP-C-N1 is an O’PROTAC designed to target the ERG-binding motif, conjugated to pomalidomide for CRBN recruitment. Figure partially created with BioRender.com.

Shao and coworkers (2021) developed an oligonucleotide-based design of PROTACs, which they called ‘O’PROTACs’, aiming for the targeted degradation of transcription factors by incorporating a TF-recognizing double-stranded oligonucleotide as the binding moiety for the TOI [25]. This approach included ligands that recruit either CRBN or VHL, using pomalidomide or VH032 respectively. The double-stranded oligonucleotides were designed using the known DNA-binding sequences for lymphoid enhancer-binding factor 1 (LEF1) (18-mer, based on the sequence 5′-TACAAAGATCAAAGGGTT-3′) (Fig. 2A) and ETS-related gene (ERG) (19-mer, based on the sequence 5′-ACGGACCGGAAATCCGGTT-3′) (Fig. 2B). In order to increase the protection from exonucleases, three extra bases were added. LEF1 is a TF that complexes with β-catenin to promote Wnt target genes [26] and is implicated in cancer cell proliferation, migration, and invasion [27]. Degradative activity showed a tight SAR dependence on E3 choice and linker architecture: LEF1 was degraded only by the VHL-based O’PROTAC with the shortest linker (OP–V1) in PC-3 prostate cancer cells (Fig. 2A), whereas longer-linker VHL variants and CRBN constructs were inactive [25]. LEF1 OP-V1 effectively and dose-dependently reduced LEF1 protein levels in PC-3 and DU145 prostate cancer cells starting around 12 h and maintaining until 48 h, with a half-maximal degradation concentration (DC_50_) of 25 nM. Importantly, this had no affect on LEF1 mRNA levels [25]. In addition, LEF1 OP-V1 inhibited the transcriptional activity of the β-catenin/LEF1 complex *in vitro* and *in vivo*, by the downregulation of β-catenin/LEF1 target gene expression (CCND1 and c-MYC) [25].

ERG degradation was explored to test the feasibility of the O’PROTAC approach for other TF. ERG is overexpressed in nearly 50% of human prostate cancers due to gene fusion [28,29]. They designed ERG O’PROTACs using an ERG-specific DNA sequence and both VHL and CRBN ligands. VHL-based ERG O’PROTACs were synthesized with high purity but were ineffective in degrading ERG in VCaP cells. While the same method yielded low purity CRBN-based O’PROTACs (probably due to the instability of pomalidomide-based ligand), they used a post-synthesis conjugation to obtain CRBN-based ERG O’PROTACs, with ERG OP-C-N1 (Fig. 2B) being particularly effective, which effectively degraded ERG protein in VCaP cells in a time- and dose-dependent manner, with a DC_50_ of 182.4 nM. Conversely, ERG degradation required CRBN recruitment (with OP-C-N1 being most effective), while VHL-based ERG O’PROTACs failed, underscoring target-specific E3/linker requirements for productive ubiquitination. *In vitro*, ERG OP-C-N1 inhibited VCaP cell growth in 3D culture and decreased their invasion ability [25].

#### 2.1.3 TF-PROTACs platform

**Fig. 3:**
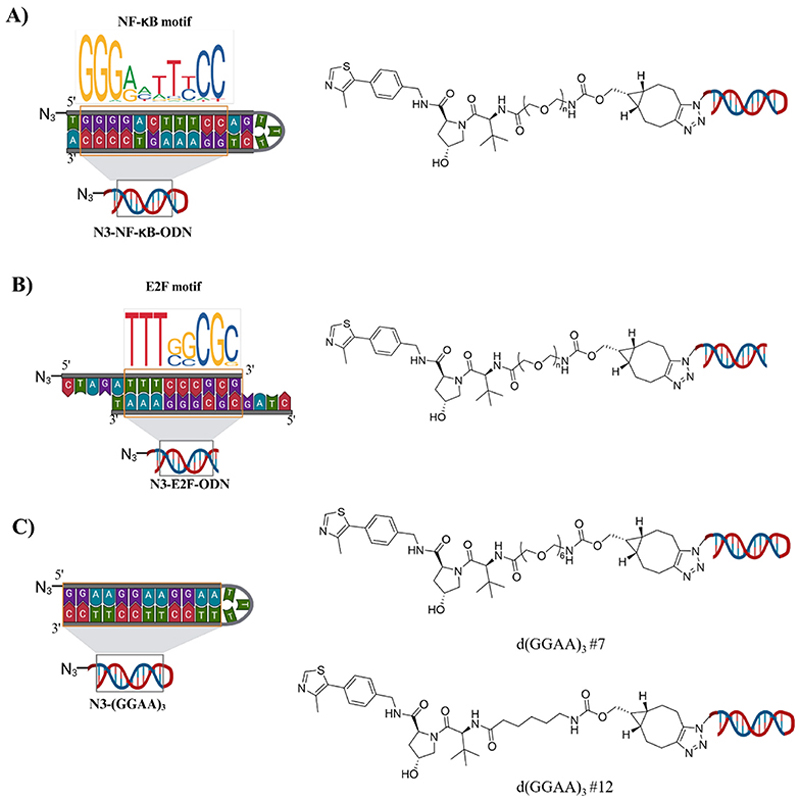
Development of TF-PROTACs. **A)** TF-PROTACs for NF-κB degradation. **B)** TF-PROTACs for E2F degradation. **C)** TF-PROTACs for degradation of ETV6 for the treatment of Ewing sarcoma, exploring PEG and alkyl linker in the design. Figure partially created with BioRender.com.

In 2021, Liu and collaborators reported the same idea of TF degradation, in which they named the approach TF-PROTACs [30]. TF-PROTACs were developed to target two transcription factors: NF-κB and E2F. NF-κB was selected because its DNA-binding motif is well-defined and validated [31]. A single-stranded DNA oligonucleotide, 5′-TGGGGACTTTCCAGTTTCTGGAAAGTCCCCA-3′ (NF-κB-ODN) (Fig. 3A), forming a double-stranded hairpin structure, was used in the design of the TF-PROTAC. Strain-Promoted Azide–Alkyne Cycloaddition (SPAAC) produced NF-κB TF-PROTACs, which utilized VH032 as E3 ligase ligand. SAR was dominated by the VHL–linker architecture: among 18 VHL-linker variants (varying length/polarity), only a small subset produced robust TF degradation, while stereochemical inversion of the key VHL hydroxyl (negative control) abolished degradation, indicating a narrow geometry window and strict dependence on functional VHL engagement. Among the resulting compounds, those bearing PEG linkers were the most effective at degrading the target protein p65 (Fig. 3A). The platform was also tested against another transcription factor, E2F1, using a double-stranded 15-mer DNA containing an E2F binding motif. The sense strand was 5′-CTAGATTTCCCGCG-3′ and the antisense strand was 5′-CTAGCGCGGAAAT-3′ (Fig. 3B). In cell-based assays, dE2F led to reduced endogenous E2F1 protein levels in HeLa cells. Overall, the TF-PROTAC platform presents a generalizable strategy for targeting “undruggable” transcription factors that have known DNA-binding motifs, potentially broadening the spectrum of druggable targets for therapeutic applications [30].

Zhu et al. (2025) designed and synthesized a novel class of TF-PROTACs for the treatment of Ewing sarcoma (Fig. 3C) [32]. In this type of cancer, EWS::FLI1 is the primary oncogenic driver in about 85% of cases, this fusion protein binds to microsatellites GGAA repeats modulating gene expression [33,34]. It was recognized that disrupting the GGAA-binding activity of EWS::FLI1 shows toxicity Ewing sarcoma due to inactivation of oncogenic gene expression [35]. Thus, EWS::FLI1 represents a good target for degradation, however no small molecules are available that binds to this fusion protein. ETV6 was identified as a vulnerability in Ewing sarcoma, as ETV6 act as a repressor of the EWS:: FLI1 transcriptional activity by its competition with EWS::FLI1 for the microsatellites GGAA [36], making ETV6 a good drug target for treating Ewing sarcoma. In the design of the TF-PROTAC, microsatellite GGAA repeats were used together with the strategy to induce the formation of double-strand hairpin to increase the stability of the compounds generated. SAR was driven by both the DNA decoy sequence and the VHL-linker geometry, (GGAA)_3_ conferred selective binding/degradation of ETV6 (not EWS::FLI1), whereas increasing the repeat to (GGAA)_4_ led to dual degradation of ETV6 and EWS::FLI1, and across 17 VHL-linker variants only a small subset produced robust ETV6 degradation, indicating a narrow, linker-dependent window for productive ubiquitination. Thus, TF-PROTACs bearing (GGAA)_3_ oligonucleotide (Fig. 3C), linker and VHL E3 ligase ligand selectively degraded endogenous ETV6 protein in Ewing sarcoma cells without significantly affecting EWS::FLI1 protein levels or other ETS family members like ERG. Selective ETV6 degradation by d(GGAA)3s leads to upregulation of known EWS::FLI1 target genes, which leads to cellular stress and DNA damage. In addition, d(GGAA)_3_s sensitize Ewing sarcoma cells to standard chemotherapy agents, which could be potential use in combination therapies to improve outcomes of Ewing sarcoma patients [32].

#### 2.1.4 STAT3 decoy–based PROTACs

**Fig. 4:**
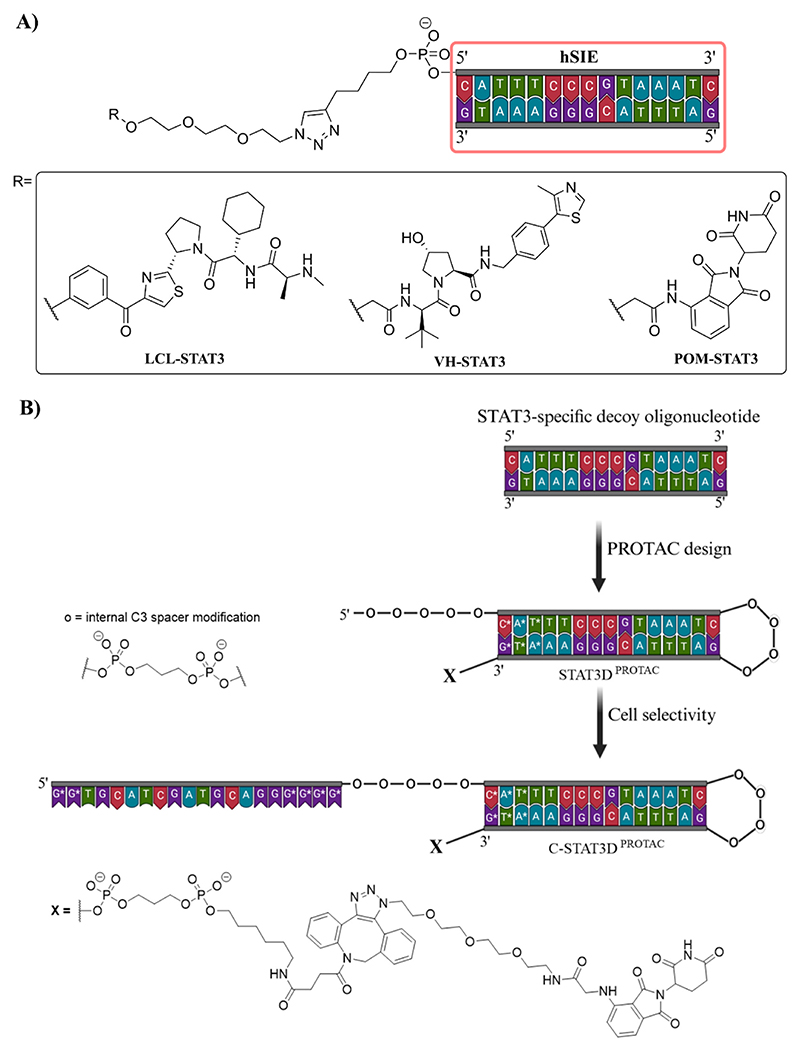
STAT3 oligonucleotide-based PROTACs. **A)** Structure of STAT3 decoy oligonucleotide-warheaded PROTAC molecules incorporating the hSIE binding motif. **B**) Structure of C-STAT3D^PROTAC^ for the cell-selective and targeted degradation of STAT3. Figure partially created with BioRender.com.

Shih et al. (2023) described the development of decoy oligonucleotide-warheaded chimeric molecules designed to target and degrade signal transducer and activator of transcription 3 (STAT3) [37]. STAT3 is a transcription factor implicated in cancer progression, including promoting cell survival, proliferation, angiogenesis, metastasis, and resistance to chemotherapy and radiation [38–40]. Efforts to develop small-molecule inhibitors of STAT3 have been hindered by the shallow and flat surface of the protein, making it a challenging drug target [41,42]. To overcome this, they explored a novel approach using oligonucleotide-based PROTACs to inhibit STAT3 activity. These STAT3-targeting PROTACs were designed by conjugating a known STAT3-specific double-stranded decoy oligonucleotide, the human serum-inducible element (hSIE) [43], to various E3 ligase ligands via alkyne–azide click chemistry [37]. The resulting chimeras included LCL-STAT3 (targeting Inhibitor of Apoptosis (IAP)), VH-STAT3 (targeting VHL) and POM-STAT3 (targeting CRBN) (Fig. 4A). SAR indicated that E3 recruiter identity was decisive: replacing VHL (VH032) or IAP (LCL161) recruiters with CRBN (pomalidomide) converted the construct into a potent degrader, with POM-STAT3 driving dose-dependent STAT3 loss (DC_50_ ~261 nM; Dmax ~78% at 1 μM), whereas VH-STAT3 was weaker and LCL-STAT3 was inactive. Scrambling the STAT3 decoy duplex (POM-Scramble) abolished degradation and excess free STAT3 decoy competed the effect, demonstrating strict sequence-dependent target engagement as a key SAR element [37]. Correspondingly, POM-STAT3 also caused a dose-dependent reduction in cell viability in NCI–H2087 cells, with a half-maximal inhibitory concentration (IC_50_) of 792 nM after 24 h of treatment. In contrast, the STAT3 decoy DNA alone showed an IC_50_ of 10 μM, indicating that targeted STAT3 degradation significantly enhances cytotoxicity compared to merely inhibiting STAT3-DNA binding [37].

Hall and co-authors (2024) described the design and evaluation of novel oligoPROTACs for the cell-selective and targeted degradation of the transcription factor STAT3 [19]. A key challenge for most oligonucleotide therapeutics is the lack of cell-selective delivery methods, which is especially important in the case of STAT3, where non-cell-selective STAT3 inhibition is undesirable because STAT3 also plays roles in non-malignant cells, including cytotoxic CD8 T cells and memory T cells, which are important for antitumor immunity [44,45]. First, STAT3PROTAC was designed based on the conjugation of STAT3-specific decoy oligonucleotide to pomalidomide (CRBN ligand) (Fig. 4B). STAT3D^PROTAC^ was shown to selectively induce CRBN-mediated proteasomal degradation of STAT3 using concentrations as low as 100 nM in mouse A20 lymphoma cells. To achieve cell selectivity, STAT3D^PROTAC^ was conjugated to a CpG oligonucleotide [46], which is a ligand for Toll-like receptor 9 (TLR9), creating C-STAT3D^PROTAC^. TLR9 is expressed on specific immune cells, including myeloid cells and B cells, as well as some cancer cells, such as B-cell lymphoma cells [47]. C-STAT3D^PROTAC^ was internalized by TLR9-positive cells like mouse A20 B-cell lymphoma, as it was able to reduce total STAT3 levels without the use of any transfection reagent, validating their design. SAR showed that linker length at the 3′ thalidomide (CRBN) attachment site was critical: a single spacer unit produced the strongest STAT3 degradation, whereas no linker (direct conjugation) or a longer three-spacer linker were less effective. Incorporating a 5′ CpG targeting domain did not diminish degradative activity, indicating that upstream delivery/targeting modules could be added without disrupting the productive CRBN–STAT3 geometry. In addition, *in vivo* treatment with C-STAT3D^PROTAC^ was more effective at reducing tumor volume than the standard C-STAT3D, providing a superior efficacy [19].

#### 2.1.5 ERα decoy–based PROTACs

**Fig. 5:**
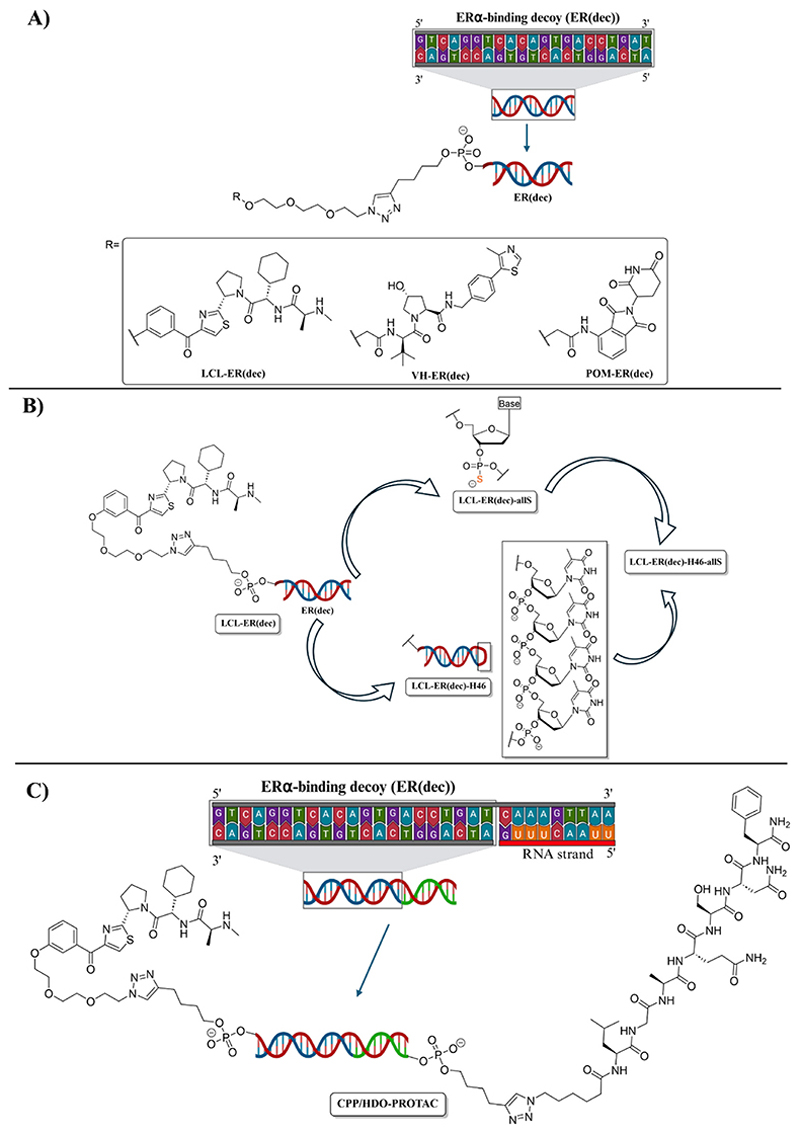
Oligonucleotide-based PROTACs targeting ERα. **A)** First class of ERα PROTACs exploring common E3 ligase ligand. **B)** Optimization of ERα PROTACs by exploring phosphorothioate (PS) modifications and hairpin structures. **C)** ERα PROTACs conjugated with hydrophobic cell-penetrating peptide (CPP) for intracellular delivery. Figure partially created with BioRender.com.

Naganuma et al. (2022) described the development of a novel type of chimeric protein degrader utilizing oligonucleotide decoys as ligands for TFs [48]. In their study, Estrogen Receptor alpha (ERα) was selected as a model TF due to its biological relevance and prior demonstration as a degradable target using small molecules [49,50] or peptides [51]. The PROTACs were synthesized by conjugating a thermodynamically stable double-stranded decoy (ER(dec)) [52] to various E3 ubiquitin ligase ligands commonly used in PROTACs: LCL161 (LCL) for IAPs, VH03 for VHL, and pomalidomide for CRBN (Fig. 5A). Conjugating the same ER (dec) duplex to different E3 recruiters revealed a clear SAR, although ERα binding affinities were comparable, the IAP ligand LCL161 conjugate (LCL-ER(dec)) produced the strongest ERα degradation, whereas VH032- and pomalidomide-conjugates were weaker. Within the LCL series, linker optimization showed a PEG3 tether gave the highest activity (vs PEG2/PEG4 or longer alkyl linkers), and ERα degradation was lost with an IAP-binding–defective N-methyl LCL control or scrambled decoy sequences, establishing recruiter competence and DNA-sequence specificity as key SAR determinants. The resulting PROTACs retained strong binding affinity to ERα, with IC_50_s below 50 nM. In ERα-positive MCF-7 breast cancer cells, LCL-ER(dec) was the most effective in reducing ERα protein levels, through a mechanism dependent on the UPS. Functionally, LCL-ER(dec) inhibited ERα-dependent transcriptional activity more effectively than the decoy alone, consistent with its degradation capability [48].

Naganuma et al. (2023) described the structural optimization of a previously developed decoy oligonucleotide-based PROTAC targeting ERα [53]. A key limitation of DNA-based PROTACs is their low intracellular stability due to susceptibility to nuclease-mediated degradation. To address this, the authors designed and synthesized new decoy-based PROTACs incorporating chemical modifications commonly used in oligonucleotide therapeutics: phosphorothioate (PS) modifications and hairpin structures (Fig. 5B). The target molecules were synthesized by conjugating an alkyne-modified sense strand of the decoy to an azide-modified LCL161 ligand using a copper-catalyzed click reaction. These novel decoy PROTACs exhibited improved binding affinity to ERα compared to the original LCL-ER(dec). The enhanced hydrophobicity from the PS modifications likely contributed to this improved binding. However, it also resulted in non-specific protein interactions, as Erα degradation was observed even with a scrambled PS-oligonucleotide-based PROTAC. Furthermore, the combination of the hairpin structure and PS modification conferred resistance to enzymatic degradation by exonuclease III. The PS-modified decoy also demonstrated significantly higher cellular uptake—19-fold greater than that of the unmodified or hairpin-only decoys—likely due to increased hydrophobicity and stronger membrane interactions [54]. PS-modified PROTACs showed higher degradation activity at much lower concentrations (0.1 μM), also displaying a hook effect, compared to natural or hairpin-only versions, which were effective at 10 μM. In summary, this work demonstrates that incorporating a hairpin structure into decoy oligonucleotide-based PROTACs enhances nuclease resistance and provides more sustained degradation activity. Although PS modifications significantly improve stability and cellular uptake, they can compromise target selectivity, highlighting a key consideration in the design of modified oligonucleotide-based PROTACs.

Building on the discovery of ERα-targeting oligonucleotide-based PROTACs, Ohaka and co-workers addressed a major challenge associated with their intracellular delivery [55]. Due to their anionic nature and chemical instability, these PROTACs typically require transfection reagents for cellular uptake, which poses significant obstacles for clinical application. To overcome these limitations, the authors designed a novel hydrophobic cell-penetrating peptide (CPP) and heteroduplex oligonucleotide (HDO)-conjugated PROTAC, termed CPP/HDO-PROTAC (Fig. 5C). This construct incorporates a hydrophobic CPP, the P4 peptide (LGAQSNF), which enhances cellular uptake [56, 57]. The design also enabled the intracellular release of the active decoy oligonucleotide-based PROTAC via RNase H-mediated cleavage of the RNA strand. The PROTAC component, LCL-HDO-F, combines a derivative of LCL161 (an IAP E3 ligase ligand) with a 30-mer DNA strand. The complementary strand, P4-HDO-R, contains a 9-mer RNA sequence and is conjugated to the P4 peptide. CPP/HDO-PROTAC demonstrated high binding affinity to ERα, with an IC_50_ of 3.0 nM, indicating that the conjugation with the P4 peptide does not compromise binding activity. It was also confirmed to be susceptible to RNase H-mediated cleavage *in vitro*. Notably, CPP/HDO-PROTAC was able to penetrate cells and induce ERα degradation at 10 μM without the need for transfection reagents. Furthermore, it inhibited the proliferation of MCF-7 human breast cancer cells under non-transfection conditions at the same concentration. This study highlights the potential of using cell-penetrating peptides and RNase H-mediated cleavage for the efficient delivery and activation of decoy oligonucleotide-based PROTACs in cells, offering a promising approach for therapeutic development [55].

#### 2.1.6 c-Myc decoy–based PROTACs

**Fig. 6:**
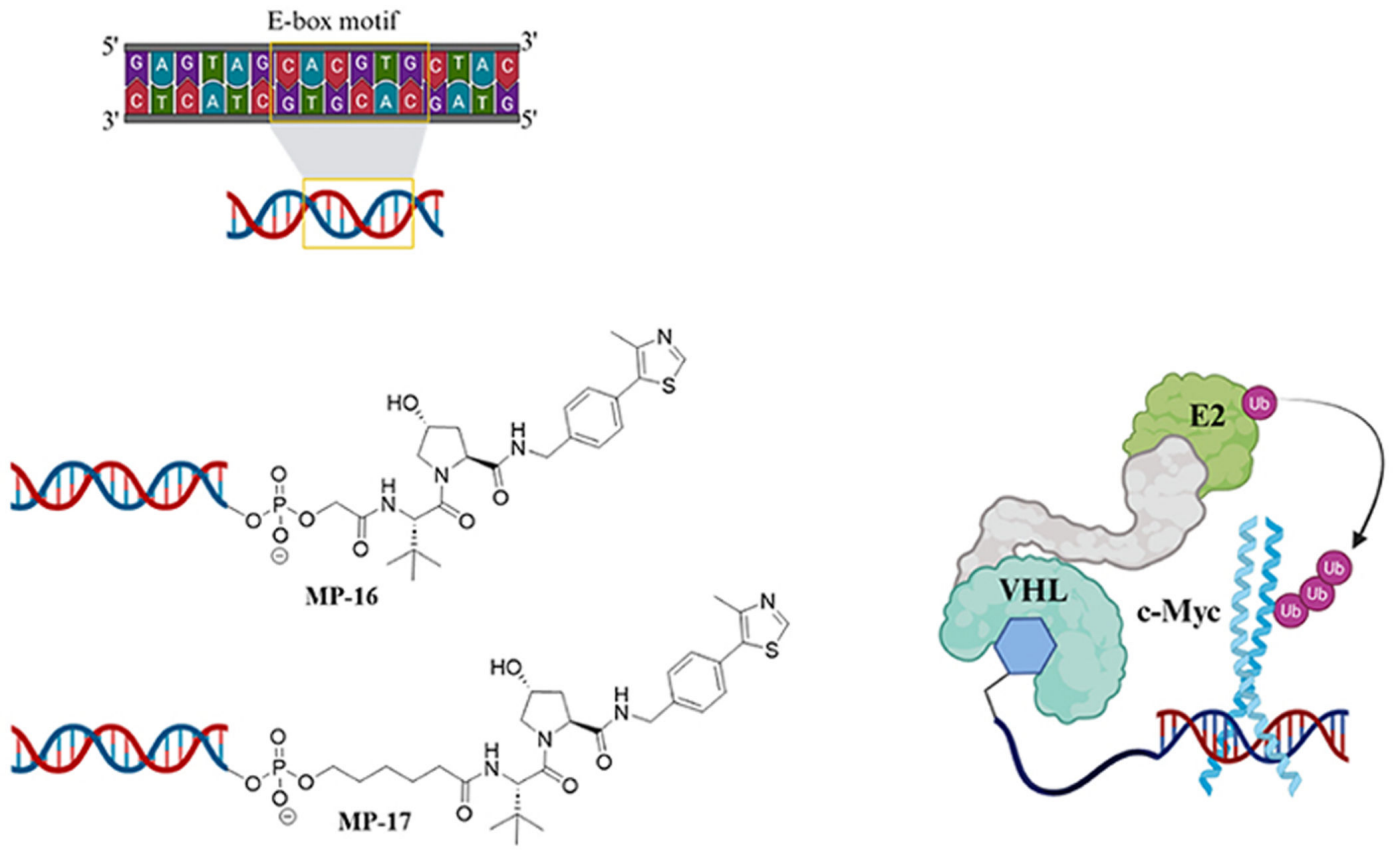
Oligonucleotide-based PROTACs for c-Myc degradation. Figure partially created with BioRender.com.

Ai et al. (2024) described novel oligonucleotide-based PROTACs, termed MP-16 and MP-17, designed to effectively induce the degradation of c-Myc for the treatment of hepatocellular carcinoma (HCC) [58]. Their design utilized an optimized DNA sequence that specifically recognizes the c-Myc complex. This optimized DNA sequence was based on a previously resolved X-ray structure of the c-Myc-Max heterodimer bound to DNA containing the E-box motif (5′-CACGTG-3′) [59]. To reduce molecular weight while maintaining affinity for the c-Myc-Max heterodimer, the DNA sequence length was further shortened. SAR was driven by E3 selection and linker geometry: a CRBN/lenalidomide series spanning multiple alkyl/PEG linkers (MP-1–MP-10) failed to degrade c-Myc, whereas VHL/VH032 conjugates enabled degradation and short-linker designs were optimal, with MP-16 and MP-17 emerging as the most active (consistent with docking-predicted spacing). In parallel, DNA warhead optimization showed that truncating the original duplex preserved c-Myc/Max binding, but E-box integrity was essential, since E-box–mutated MP-16/MP-17 lost degradative activity. MP-16 and MP-17 (Fig. 6) directly interact with the c-Myc complex to form a VHL/PROTAC/c-Myc ternary complex. This complex recruits the ubiquitin-proteasome system, triggering c-Myc degradation. In HCC cell lines (SK-Hep-1 and SNU-398), MP-16 and MP-17 effectively degraded c-Myc protein. The optimal degradation conditions were determined to be at a concentration of 0.4 μM and 8 h after transfection. Mutation of the E-box motif in MP-16 and MP-17 abolished their c-Myc degrading activity, highlighting the critical role of this motif. MP-16 and MP-17 significantly suppressed the proliferation of HCC cells *in vitro* in a dose-dependent manner, as demonstrated by MTT and colony formation assays. To evaluate their efficacy *in vivo*, MP-16 and MP-17 were encapsulated in nanoparticles to facilitate drug delivery and administered intravenously to mice bearing HCC xenograft tumors, inhibiting tumor growth [58].

#### 2.1.7 RNA-PROTACs

**Fig. 7:**
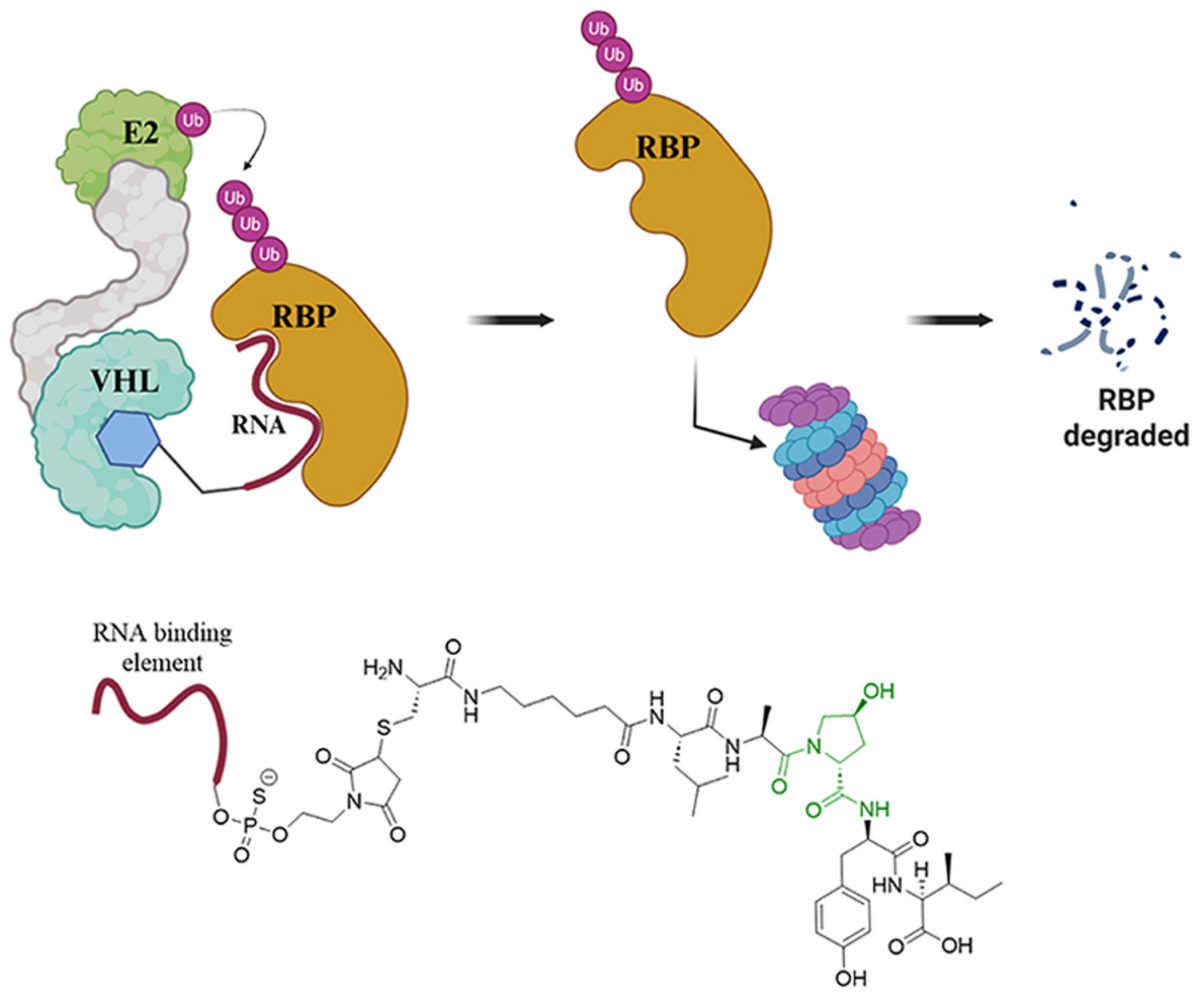
The development of RNA-PROTACs, in green is highlighted the hydroxylated proline derived from HIF-1α protein. Figure partially created with BioRender.com.

Ghidini et al. (2021) developed a novel strategy called RNA-PROTACs for targeting and degrading RNA-Binding Proteins (RBPs) [60]. RBPs are involved in several diseases, however targeting them with small molecule drugs has been challenging, due to intrinsic disorder of RBP binding pockets and the change in conformation upon RNA binding [61,62]. Like conventional PROTACs, which use a small molecule to bind the target protein, RNA-PROTACs comprise an RBP-binding moiety linked to an E3 ubiquitin ligase-recruiting ligand that induce the selective degradation of a target RBP via the cell’s ubiquitination and proteasome machinery (Fig. 7). As a proof-of-concept, RNA-PROTACs targeting LIN28 (Lin28A) were developed. LIN28 is an RBP that acts as a stem cell factor and oncoprotein, which present a cold-shock domain (CSD) and a zinc knuckle domain (ZKD), composed of two Cys-Cys-His-Cys (CCHC) zinc finger domains [61]. 7-nucleotide sequence (5′-AGGAGAU-3′) that is a conserved Lin28 binding element present in microRNAs was used as a ligand. To ensure stability and cellular uptake, they incorporated structural modifications, such as PS linkages and 2′-*O*-methoxyethyl (MOE) ribonucleotides [63–66], which did not disturb the oligonucleotide’s binding interactions with the Lin28 ZKD domain. An E3-recruiting peptide derived from the HIF-1α protein (specifically a shortened segment containing a hydroxylated proline; LA [Hyp]YI) was conjugated to the modified oligonucleotides (Fig. 7). These RNA-PROTACs, including ORN3P1 (using a PS-MOE 7-mer oligonucleotide and the LA[Hyp]YI peptide) and ORN7P1 (using an 11-mer), were shown to bind to Lin28A, to mediate ubiquitination of Lin28A in cells and to lead to proteasomal degradation of Lin28A. The approach was also used in other RBPs, such as RBFOX1 - a splicing factor - [67] showing that the strategy can be generalizable. SAR indicated that oligonucleotide chemistry and recruiter competence-controlled degradation, converting the short Lin28/RBFOX1 RNA-binding element to phosphorothioate analogs markedly improved functional competition/engagement and enabled ~50% target knockdown, whereas the unmodified phosphodiester 7-mer was inactive. Degradation remained strictly sequence- and E3-dependent, since randomized RBEs or a VHL-recruiting peptide control lacking hydroxyproline abolished activity, and a VH032-conjugated variant also supported proteasome-dependent Lin28A loss. This establishes RNA-PROTACs as a promising new concept for targeting RBPs, a protein class that has been challenging to address with conventional drugs [60].

### 2.2 Aptamer-based PROTACs

Aptamers are single-stranded DNA or RNA oligonucleotides capable of binding to targets with high affinity and specificity, while exhibiting minimal immunogenicity and toxicity [68,69]. Aptamers are usually identified through Systematic Evolution of Ligands by Exponential Enrichment (SELEX), which screens random oligonucleotide sequences for their ability to bind a particular target [70]. Aptamer-based PRO-TACs utilize a target-binding aptamer, as POI ligand, providing high-affinity and highly selective recognition of the POI. Considerations need to be taken related with the linker to prevent any disruption of the aptamer. In this session, we describe examples that explore this design.

#### 2.2.1 AS1411-based PROTACs

**Fig. 8:**
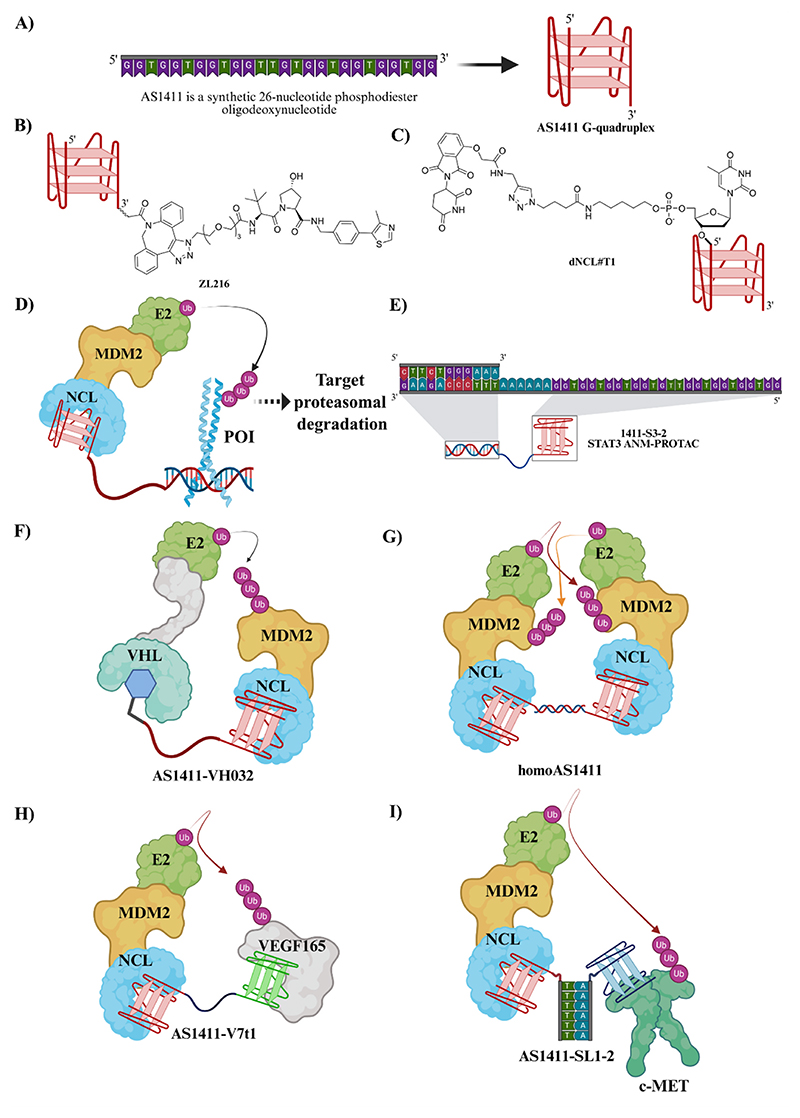
AS1411 as a versatile aptamer in target protein degradation. **A)** Illustration of the structure of AS1411, a guanine-rich 26-mer DNA aptamer that forms a G-quadruplex conformation. **(B)** Structure of NCL degrader ZL216. **(C)** Structure of NCL degrader dNCL#T1. **(D)** AS1411 acts as an ‘E3 ligase ligand’ by binding to nucleolin (NCL), facilitating the recruitment of MDM2 and resulting in proteasomal degradation of the POI. **(E)** Structure of STAT3 degrader 1411-S3-2. **(F)** Scheme of the proteasomal degradation of MDM2 through AS1411-VH032 **(G)** Scheme of the proteasomal degradation of MDM2 through homoAS1411 **(H)** Scheme of the proteasomal degradation of VEGF165 through AS1411-V7t1. **(I)** Scheme of the proteasomal degradation of c-MET through AS1411-SL1-2. Figure partially created with BioRender.com.

AS1411 is a synthetic 26-nucleotide unmodified DNA aptamer with the sequence 5′-GGTGGTGGTGGTTGTGGTGGTGGTGG-3′ (Fig. 8A). AS1411 is rich in guanine and forms a G-quadruplex structure [71], which enables selective binding to nucleolin (NCL), a multifunctional protein overexpressed on the surface of many tumor cells, but largely absent from the surface of normal cells [72–75]. NCL is predominantly located in the nucleolus, where it contributes to critical cellular functions such as ribosome biogenesis, chromatin remodeling, transcriptional regulation, and cell proliferation [73,76]. However, in cancer cells, NCL is often upregulated and mislocalized to the cell surface [77, 78], making it an attractive therapeutic target. Importantly, AS1411 not only binds specifically to surface-expressed NCL but also exploits it as a route for internalization. Upon binding, the AS1411–NCL complex is internalized into tumor cells, where it can exert cytostatic and cytotoxic effects, disrupting essential processes and inhibiting tumor growth [79]. This dual role, targeting a cancer-specific marker and enabling intracellular delivery, positions NCL as a promising entry point for aptamer-based drug delivery and cancer therapy, which can be also exploited in the TPD.

AS1411 has progressed through Phase II trials, including metastatic renal cell carcinoma [80] and acute myeloid leukaemia [81], showing excellent safety profile and durable response in some patients. However, the suboptimal pharmacology and low potency limited further development of AS1411 [75]. In this session, we present case studies demonstrating the potential use of AS1411 in TPD, where it functions both as a POI ligand and as an E3 ligase ligand.

Zhang et al. described the development of ZL216, [82] a novel PROTAC for the tumor-selective degradation of NCL. ZL216 was created by conjugating the AS1411 to a small-molecule ligand that recruits the E3 ubiquitin ligase VHL (Fig. 8B). ZL216 retained the binding specificity of AS1411, selectively binding to breast cancer cells (MCF-7 and BT474), but not to normal breast epithelial cells (MCF-10A). ZL216 demonstrated high binding affinity to breast cancer cells, with dissociation constants in the nanomolar range (*K*_d_ = 12 nM in MCF-7 and *K*_d_ = 15 nM in BT474). ZL216 was shown to downregulate NCL levels in MCF-7 and BT474 breast cancer cells both *in vitro* and *in vivo*. Furthermore, ZL216 treatment inhibited the proliferation and migration of MCF-7 breast cancer cells *in vitro*, while having no effect on normal breast cells (MCF-10A). Notably, ZL216 exhibited activity at nanomolar concentrations, compared to AS1411, which required micromolar concentrations—highlighting the catalytic mode of action of ZL216 [82].

In an independent work, Chen and coworkers (2023) developed a novel approach for targeted protein degradation using aptamer-based PROTACs (apt-PROTACs) [83]. They chose NCL, as a proof-of-concept target. A series of NCL degraders, termed dNCL, were generated by conjugating a CRBN ligand to the AS1411 aptamer. Through linker optimization, the molecule dNCL#T1 (Fig. 8C), which possesses thymidine as a linker, was identified as the most effective NCL degrader. dNCL#T1 binds to NCL with a dissociation constant (*K*_d_) of 0.36 μM. In addition, treatment with dNCL#T1 led to the ubiquitination of NCL and its proteasome-dependent degradation. NCL degradation started at 12h with a concentration as low as 125 nM. dNCL#T1 demonstrated significant antitumor efficacy in breast cancer cell lines (MCF-7, MDA-MB-231), which express elevated levels of NCL. Treatment with dNCL#T1 suppressed cell proliferation, inhibited 3D spheroid growth, and significantly attenuated cell migration. As previously observed [80], treatment with the apt-PROTAC was effective at a lower dose (0.5 μM) when compared to the AS1411 aptamer alone, which required high doses (10 μM) to produce similar effects [82]. In order to avoid potential on-target toxicity, the authors developed a light-controllable apt-PROTAC, termed opto-dNCL#T1. This molecule was designed by hybridizing dNCL#T1 with a complementary photolabile oligonucleotide (CP). The opto-dNCL#T1 was inert until activated by UVA light. Irradiation restored the ability of opto-dNCL#T1 to promote the NCL-CRBN interaction and triggered proteasome-mediated NCL degradation [83].

In addition to the effects as a cancer-specific biomarker and enabling intracellular delivery of oligonucleotide-based drugs, NCL was found be bind to Mouse double minute 2 homolog (MDM2) [84,85]. MDM2 is a well-known E3 ligase utilized in the design of PROTACs [86,87]. Thus, it was proposed to repurpose AS1411 as a recruiter of MDM2, by anchoring the NCL–MDM2 complex, leading to targeted protein degradation [88]. Fu et al. described the development of an AS1411–NCL–MDM2-based PROTAC (ANM-PROTAC) [88]. The ANM-PROTAC platform was tested against several “undruggable” oncogenic proteins, including transcription factors (STAT3 and c-Myc), an androgen receptor splice variant (AR-V7), and the p53-R175H hotspot mutant. Each ANM-PROTAC was constructed by conjugating AS1411 with a ligand specific to the POI. The resulting ANM-PROTACs efficiently degraded their respective oncogenic targets—STAT3, c-Myc, AR-V7, and p53-R175H—via the ubiquitin–proteasome pathway. This degradation was dependent on both NCL and MDM2. Furthermore, ANM-PROTACs exhibited tumor cell selectivity, degrading targets such as STAT3 in tumor cells but not in non-malignant cells. They also significantly inhibited tumor growth and induced apoptosis *in vitro*. The optimized STAT3 degrader, 1411-S3-2 (Fig. 8D), demonstrated a lower DC_50_ value (139 nM) compared to a previously reported small-molecule STAT3 PROTAC (3 μM). *In vivo* studies in xenograft mouse models showed that 1411-S3-2 effectively localized to tumor sites, co-localizing with NCL and MDM2, leading to tumor-specific STAT3 degradation. This targeting resulted in tumor growth inhibition and apoptosis. Overall, this strategy demonstrates strong potential for the development of large-molecular-weight, tumor-selective PROTACs for cancer therapy [88].

Wang and co-authors designed novel MDM2-targeting PROTAC degraders with built-in tumor-targeting capabilities using the AS1411 aptamer [89]. Two AS1411-based MDM2 degraders were developed: AS1411-VH032 (Fig. 8F) – a PROTAC created by conjugating AS1411 with VH032 (VHL ligand); homoAS1411 – a homo-PROTAC constructed by linking two AS1411 molecules (Fig. 8G), designed to promote “suicide” degradation of MDM2 via self-ubiquitination. AS1411-VH032 effectively bridged the NCL–MDM2 complex with VHL to form a quaternary structure, facilitating MDM2 ubiquitination and subsequent proteasomal degradation in a time- and concentration-dependent manner, leading to stabilization of p53. Similarly, homoAS1411 induced MDM2 degradation and p53 stabilization through MDM2 self-ubiquitination, a process also dependent on NCL. Both PROTACs showed tumor cell specificity. Neither AS1411-VH032 nor homoAS1411 bound to non-tumorigenic MCF-10A cells, which lack surface NCL, and therefore did not induce MDM2 degradation or inhibit their growth. *In vitro*, both AS1411-VH032 and homoAS1411 inhibited tumor cell proliferation, reduced colony formation, promoted apoptosis, and suppressed 3D spheroid growth across various tumor cell lines. *In vivo* treatment reduced tumor volume. Furthermore, while both AS1411-VH032 and homoAS1411 were shown to degrade NCL, this effect was observed only at significantly higher concentrations than those required for MDM2 degradation. Specifically, AS1411-VH032 induced NCL degradation at concentrations above 1000 nM, whereas effective MDM2 degradation occurred below 100 nM. Similarly, homoAS1411 required over 2500 nM to reduce NCL levels, compared to under 250 nM for MDM2 degradation. Linker length was found to influence NCL degradation; a longer linker in homoAS1411 enhanced NCL degradation but abolished MDM2 degradation, emphasizing the importance of NCL’s role as a molecular bridge in these PROTACs.

AS1411 was also used the design of novel aptamer-PROTACs to selectively degrade VEGF165 in both tumor and vascular endothelial cells [90]. VEGF165, the predominant and most potent isoform of VEGFA, is a key factor promoting tumor survival, proliferation, metastasis, and angiogenesis, making it an attractive target for antitumor strategies [91]. AS1411 was conjugated with V7t1, a single-stranded DNA (ssDNA) aptamer that specifically binds to VEGF165 [92]. These aptamers were conjugated using a six-adenine (A_6_) ssDNA linker, which preserved their G-quadruplex structures, to generate two hybrid PROTAC molecules: AS1411-V7t1 (with AS1411 at the 5′ end of V7t1) and V7t1-AS1411 (with AS1411 at the 3′ end of V7t1) (Fig. 8H). These hybrid PROTACs facilitated selective cellular internalization, bringing the NCL–MDM2 complex into proximity with VEGF165, thereby promoting its degradation. AS1411-V7t1 and V7t1-AS1411 exhibited strong binding and cellular uptake in NCL-expressing cells, but not in MCF10A cells. In HeLa and HUVEC cells, both PROTACs effectively degraded VEGF165 in a concentration- and time-dependent manner. This degradation was dependent on NCL and MDM2 expression, as well as proteasome activity. AS1411-V7t1 demonstrated greater potency than V7t1-AS1411, with DC_50_ values of 109 nM and 225 nM, respectively, in HeLa cells. The PROTACs inhibited the proliferation and migration of tumor cells (HeLa and HT29) and induced apoptosis, while showing no cytotoxic effects on MCF10A cells, consistent with the absence of NCL expression. AS1411-V7t1 also exhibited greater serum stability, leading to its *in vivo* evaluation. In mouse models, AS1411-V7t1 inhibited tumor growth, which was associated with VEGF165 degradation, reduced expression of the endothelial marker CD31, decreased tumor cell proliferation, and increased apoptosis within the tumor [90].

Fu and colleagues developed novel dual aptamer-functionalized PROTACs for the targeted degradation of c-MET in the treatment of osteosarcoma utilizing AS1411 to recruit MDM2 [93]. Existing c-MET inhibitors and degraders are limited by issues such as acquired resistance and lack of tumor specificity, leading to compromised efficacy and safety concerns [94,95]. SL1, a DNA aptamer specific for c-MET, was utilized for the design of novel aptamer-PROTACs. In addition, AS1411 was employed as a tumor-targeting ligand and as a recruiter of the E3 ubiquitin ligase MDM2. Notably, this study extends the PROTAC strategy to target a membrane protein, which is typically challenging. The AS1411-SL1 chimeras were constructed by linking the two aptamers via nucleic acid linkers (Fig. 8I). These double-stranded DNA linkers were composed of adenine-thymine (A–T) base pairs, as guanine-cytosine (G–C) pairs could interfere with the optimal formation of AS1411’s G-quadruplex structure. These PROTACs selectively degraded c-MET by facilitating the formation of an MDM2–NCL–PROTAC–c-MET complex, effectively recruiting MDM2 into proximity with c-MET via NCL acting as a molecular bridge. Functionally, the chimeras modulated phosphorylated c-MET (p-*c*-MET) levels at lower concentrations (100–200 nM), as reported [96], while higher concentrations (≥500 nM) were required to achieve degradation of total c-MET. In osteosarcoma (OS) xenograft mouse models, AS1411-SL1-2 led to inhibition of tumor growth in both subcutaneous and orthotopic OS models. Overall, this study represents a significant advancement in the development of tumor-targeted PROTACs for membrane proteins, demonstrating the feasibility and therapeutic potential of a dual-aptamer strategy [93].

#### 2.2.2 Aptamer-based PROTACs targeting p53-R175H

**Fig. 9:**
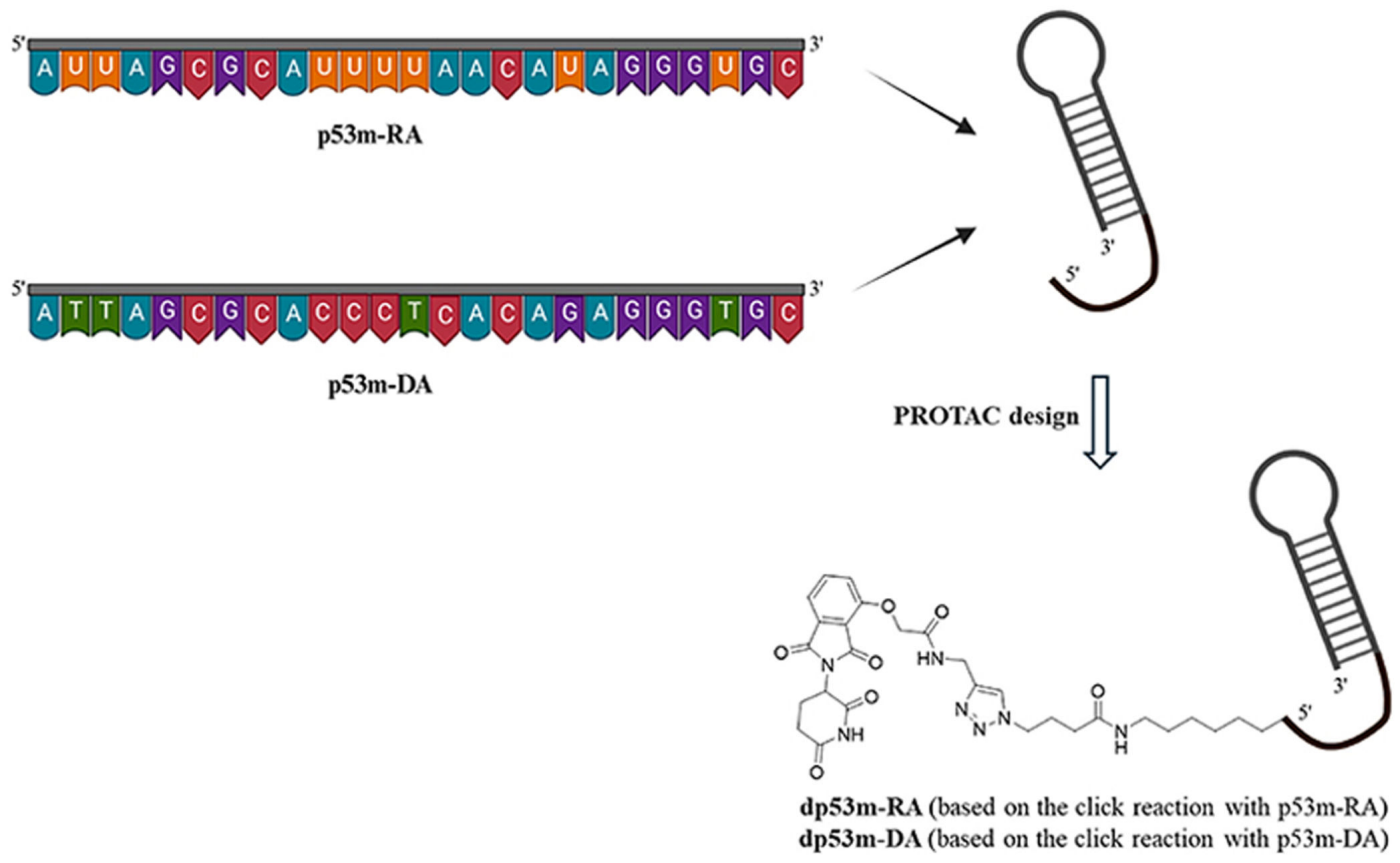
Design of aptamer-based chimeric molecules for targeted protein degradation of p53-R175H. Figure partially created with BioRender.com.

Kong et al. (2022) reported the development of the first selective PROTAC targeting the oncogenic p53-R175H hotspot mutant — the most common p53 mutation, known to drive cancer progression by promoting cell proliferation, migration, and invasion [97,98]. Although p53-R175H is a critical cancer-related target, it has proven difficult to drug, even with PROTACs, due to the lack of suitable small-molecule binders. To overcome this limitation, an RNA aptamer (p53m-RA (5′-AUUAGCGCAUUUUAACAUAGGGUGC-3′)) that selectively binds to p53-R175H was used (Fig. 9) [99]. A p53-R175H-targeting degrader named dp53m-RA was designed and synthesized (Fig. 9) by conjugating an alkynylated CRBN ligand (thalidomide-*O*-amido-propargyl) to the 5′ end of N_3_-p53m-RA via a click reaction. dp53m-RA selectively degraded p53-R175H in p53-null H1299 cells engineered to express the mutant. Degradation occurred in a dose-dependent manner, with a DC_50_ of approximately 1 μM across different cell lines, and was observable within 12 h of treatment [98]. Notably, dp53m-RA specifically degraded p53 molecules with R175H but not with the other tested hotspot mutations. Moreover, treatment with dp53m-RA upregulated the expression of several downstream p53 effectors — including MDM2, BAX, CDKN1A, and PUMA — in cells expressing p53-R175H, but not in cells expressing wild-type p53 [98].

RNA aptamer p53m-RA exhibits limited clinical utility due to its instability in serum [98]. Thus, a high-performance DNA aptamer targeting the p53-R175H mutant was developed [100]. Using an iterative, molecular docking-guided post-SELEX approach, a more stable DNA variant was designed, termed p53m-DA (Fig. 9) [100]. Molecular dynamics simulations provided insights into the structural differences between wild-type p53 (p53-WT) and the mutant p53-R175H. p53m-DA showed significantly improved serum stability and a higher affinity for p53-R175H, with a dissociation constant (*K*_d_) of 0.29 μM. It also demonstrated 14-fold selectivity over p53-WT. To enable targeted degradation, a PROTAC molecule, dp53 m, was synthesized (Fig. 9) by using the same approach from their previous work [98]. dp53 m induced dose- and time-dependent degradation of p53-R175H in multiple cancer cell lines carrying the mutation (H1299-p53-R175H, Detroit 562, and SKBR3). Furthermore, dp53 m showed specificity for p53-R175H. dp53 m effectively inhibited proliferation, migration, and 3D spheroid growth of p53-R175H-driven cancer cells, while exerting minimal effects on cells expressing p53-WT. In a Detroit 562 xenograft mouse model, intravenously administered dp53 m suppressed tumor growth. Lastly, dp53 m showed a synergistic effect with cisplatin in reducing the viability of p53-R175H-expressing cancer cells, which are typically associated with chemotherapy resistance [101]. Thus, dp53 m is a promising therapeutic candidate for cancers driven by the p53-R175H mutation [100].

#### 2.2.3 Aptamer-based PROTACs targeting c-Myc

**Fig. 10:**
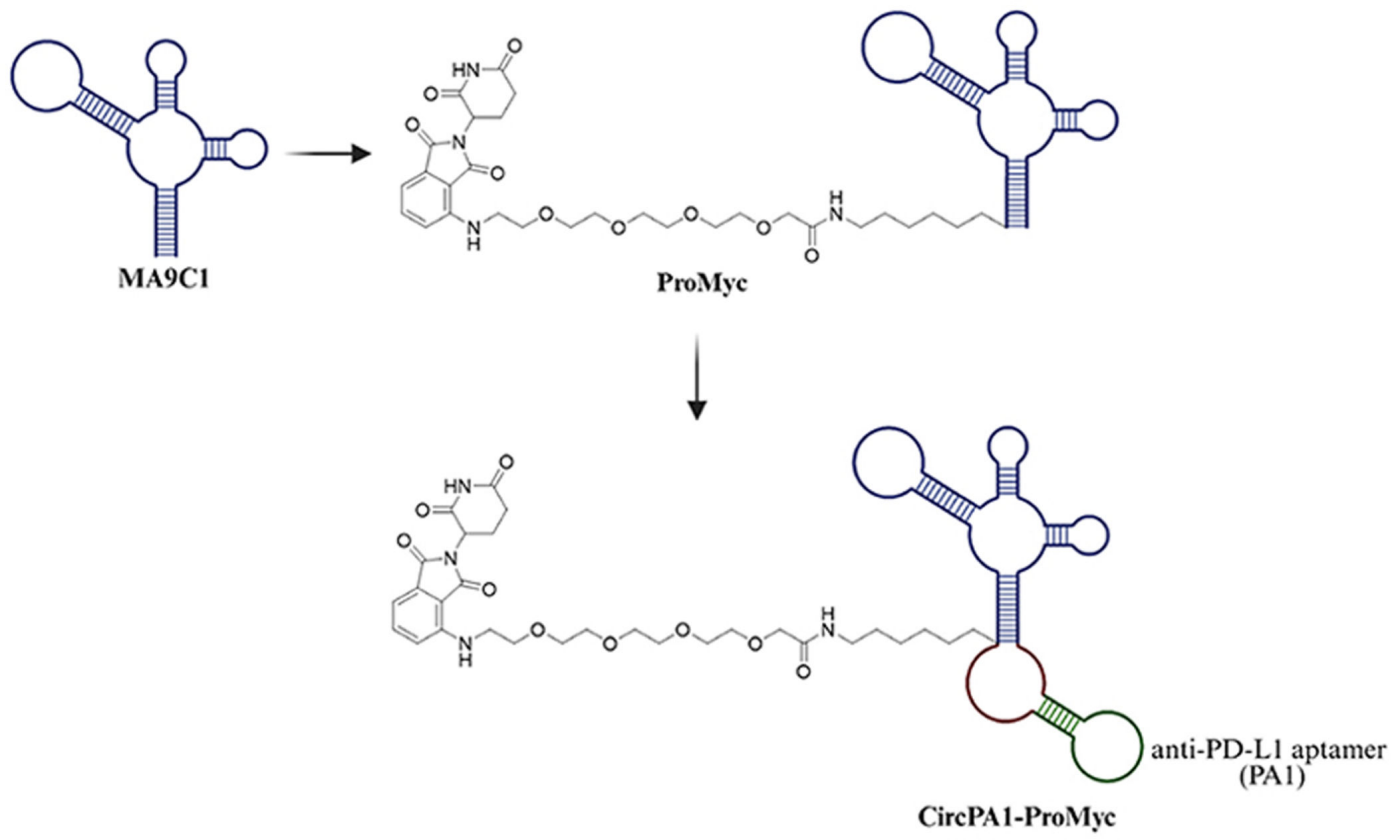
Design of aptamer-based chimeric molecules for targeted protein degradation of c-Myc. Figure partially created with BioRender.com.

Wang and colleagues described a novel approach for the targeted degradation of the c-Myc protein using an aptamer-based PROTAC [102]. Microwell-SELEX, a high-throughput screening method called ‘polystyrene microwell plate-based Systematic Evolution of Ligands by EXponential enrichment’, was used to identify MA9C1, a c-Myc-specific aptamer with high binding affinity (*K*_d_ of 29.90 nM) and strong selectivity was identified. MA9C1 was found to bind to an intrinsically disordered region of c-Myc, without interfering with c-Myc/Max dimerization or DNA binding. Based on the structure of MA9C1, they designed a novel PROTAC named ProMyc by conjugating MA9C1 to pomalidomide (Fig. 10). ProMyc exhibited enhanced stability compared to the standalone aptamer, likely due to the pomalidomide moiety protecting it from 5′ exonuclease degradation. *In vitro*, ProMyc effectively degraded endogenous c-Myc in multiple cancer cell lines (HCT116, A549, HeLa, MDA-MB-231) in a dose- and time-dependent manner, achieving up to 95% degradation and a DC_50_ of 5.02 nM in HCT116 cells. Treatment with ProMyc inhibited cancer cell proliferation, migration, and tumorigenesis. To enhance the pharmaceutical potential of ProMyc, a second-generation chimera was designed, circPA1-ProMyc, incorporating cyclization for improved stability and an anti-PD-L1 aptamer (PA1) for targeted delivery to PD-L1-positive tumor cells. CircPA1-ProMyc effectively entered cells and degraded c-Myc, while also functioning as a dual aptamer-based PROTAC, simultaneously degrading both c-Myc and PD-L1. *In vivo* studies in HCT116 colorectal cancer xenograft models demonstrated that circPA1-ProMyc significantly attenuated tumor growth and induced c-Myc degradation, while maintaining favorable safety and low immunogenicity.

#### 2.2.4 C-PROTAC platform

**Fig. 11:**
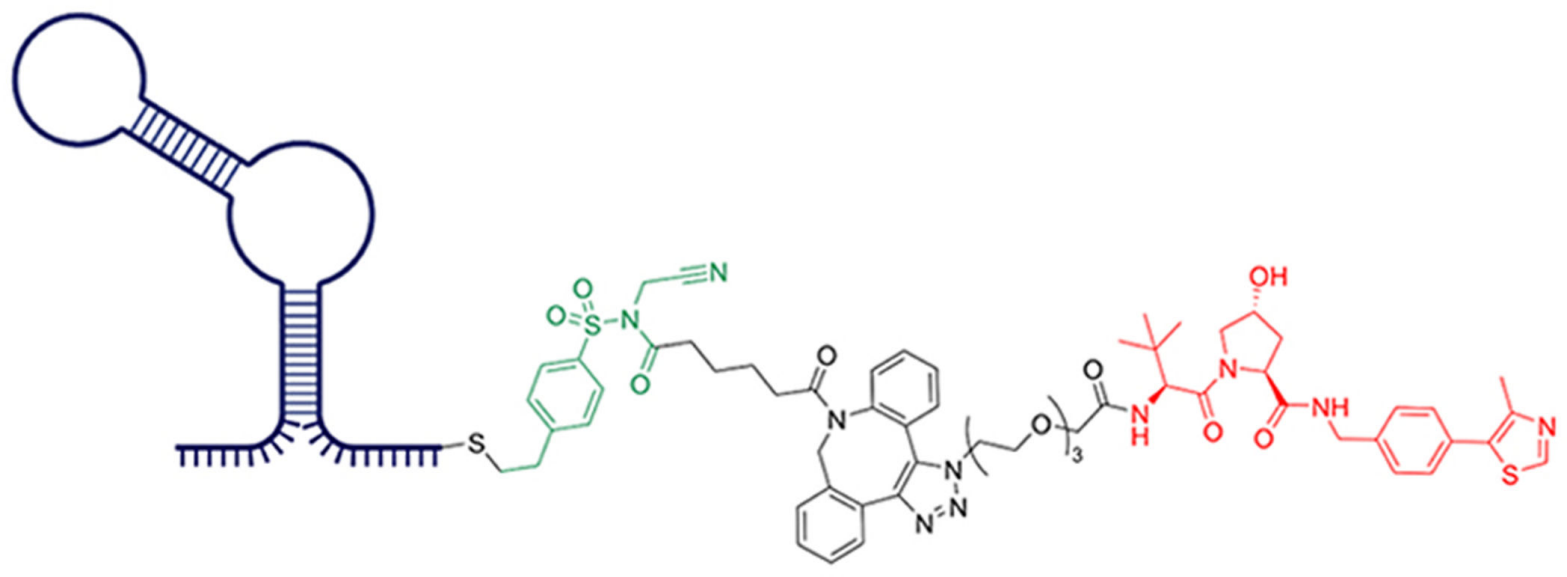
Design approach of a novel C-PROTAC for the targeted degradation of ZBP1. NASA is highlighted in green and the VHL ligand in red. Figure partially created with BioRender.com.

Huang and colleagues designed a novel covalent PROTAC (C-PROTAC) for the targeted degradation of Z-DNA binding protein 1 (ZBP1), a pathogen-sensing protein that, when activated, triggers necroptotic signaling cascades leading to strong inflammatory responses and potential tissue damage [103]. Selectively inhibiting or degrading ZBP1 represents a promising strategy to control inflammation. To degrade ZBP1, a C-PROTAC was developed by incorporating ZBP1-specific DNA aptamers, *N*-acyl-*N*-alkyl sulfonamides (NASAs) for covalent binding, a linker, and a VHL ligand (Fig. 11). SELEX was used to identify aptamers, with the top candidate, aptamer Z3, demonstrating a binding affinity of 2.71 nM, which was higher than classical Z-DNA (*K*_d_ = 47.35 nM). Covalent bond formation with ZBP1 was confirmed via mutagenesis and fluorescence labeling. Compared to a non-covalent PROTAC, the C-PROTAC achieved over a threefold improvement in ZBP1 degradation and exhibited potent, dose-dependent activity (DC_50_ = 25.69 nM). In an H1N1 influenza virus model, the C-PROTAC reduced virus-induced ZBP1 expression to levels observed in uninfected cells, significantly improved cell viability (95.8% vs. 34.9% in the inflammation model), and restored cellular proliferation. Furthermore, the C-PROTAC markedly suppressed the expression of proinflammatory cytokines including IL-18, IL-6, IL-1β, TNF-α, and IFN-β in infected cells. In a mouse model of influenza A virus (IAV) infection, C-PROTAC treatment led to a significant increase in survival (80% vs. 20% in untreated infected mice), improved body weight, and reduced lung inflammation and edema. This innovative strategy by combining aptamer specificity and covalent binding, and PROTAC-mediated degradation offers a promising therapeutic approach for targeting ZBP1 in inflammatory diseases [103].

## 3 Oligonucleotides as linkers in the design of PROTACs

**Fig. 12:**
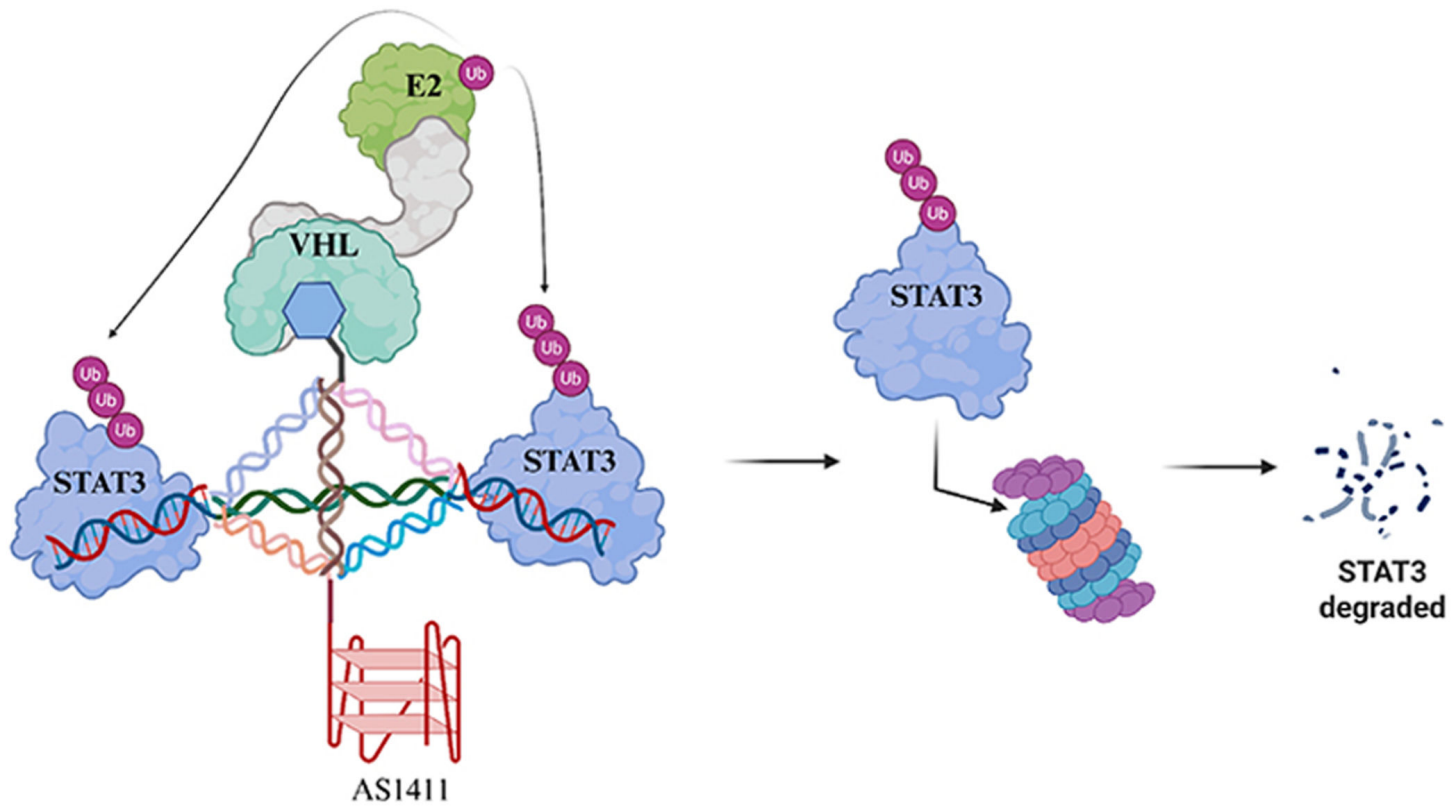
Design of DNA tetrahedron-driven multivalent PROTACs for degradation of STAT3. Figure created with BioRender.com.

Beyond their utility as POI ligand, oligonucleotides can also function in linker design. Li and coworkers developed a novel type of multivalent PROTAC, which employs a DNA tetrahedron as a linker to enhance protein degradation efficiency and tumor targeting in cancer treatment [104]. They utilized the rigidity of double-stranded DNA linkers to better constrain the spatial relationship between the target protein and the E3 ubiquitin ligase. This rigidity reduces conformational flexibility, thereby improving the accuracy of target recognition, degradation efficiency, and selectivity. AS-TD2-PRO is a DNA tetrahedron-based multivalent PROTAC with different functional components at its vertices. These include a guidance module consisting of the AS1411 aptamer, which targets NCL overexpressed on tumor cell membranes and enables tumor-specific delivery, and recognition modules composed of two STAT3 decoys and an E3-ligase ligand that recruits the VHL E3 ligase (Fig. 12). The DNA tetrahedron with a 10-base-pair edge length was found to be optimal for STAT3 degradation. AS-TD2-PRO was specifically taken up by MCF-7 breast cancer cells, which are NCL-positive. It effectively degraded STAT3 protein in these cells in a concentration-, time-, and proteasome-dependent manner, achieving over 90% degradation at a 1 μM concentration after 12 h of treatment. Compared to the monovalent AS-TD1-PRO (with a single STAT3 recognition module) and the traditional bivalent D10-PRO (lacking tumor-targeting specificity), AS-TD2-PRO demonstrated superior efficacy in degrading STAT3. It also inhibited tumor cell migration and invasion. In functional assays, AS-TD2-PRO showed enhanced antiproliferative effects in colony formation and CCK8 assays, with an IC_50_ of 993 nM in MCF-7 cells. *In vivo*, AS-TD2-PRO achieved a tumor growth inhibition rate of 95.79%, outperforming both AS-TD1-PRO and D10-PRO. DNA tetrahedron-driven multivalent PROTACs like AS-TD2-PRO represent a promising strategy for enhancing tumor-specific targeting, improving protein degradation efficiency, and achieving superior antitumor effects [104].

Recently, the concept of DNA-templated spatially controlled Proteolysis Targeting Chimeras (DTACs) was introduced by Zheng et al. (2025) [105]. In this approach, the E3 ligase ligand and the POI ligand are integrated into a DNA duplex scaffold, which provides precise control over the spacing and orientation between the two ligands. This system offers flexibility in tuning both distance and orientation, enabling multiple configurations. The Cyclin D1–CDK4/6 complex was used as a model to test the feasibility of this strategy, and the results demonstrated that degradation efficiency is dependent on both the distance and orientation between the ligands [105]. Thus, this approach highlights the promising potential of using oligonucleotides as linkers, allowing for the rational selection of optimal spacing and orientation between the E3 ligase and POI ligands.

## 4 Oligonucleotides as E3 ligase ligands in the design of PROTACs

**Fig. 13:**
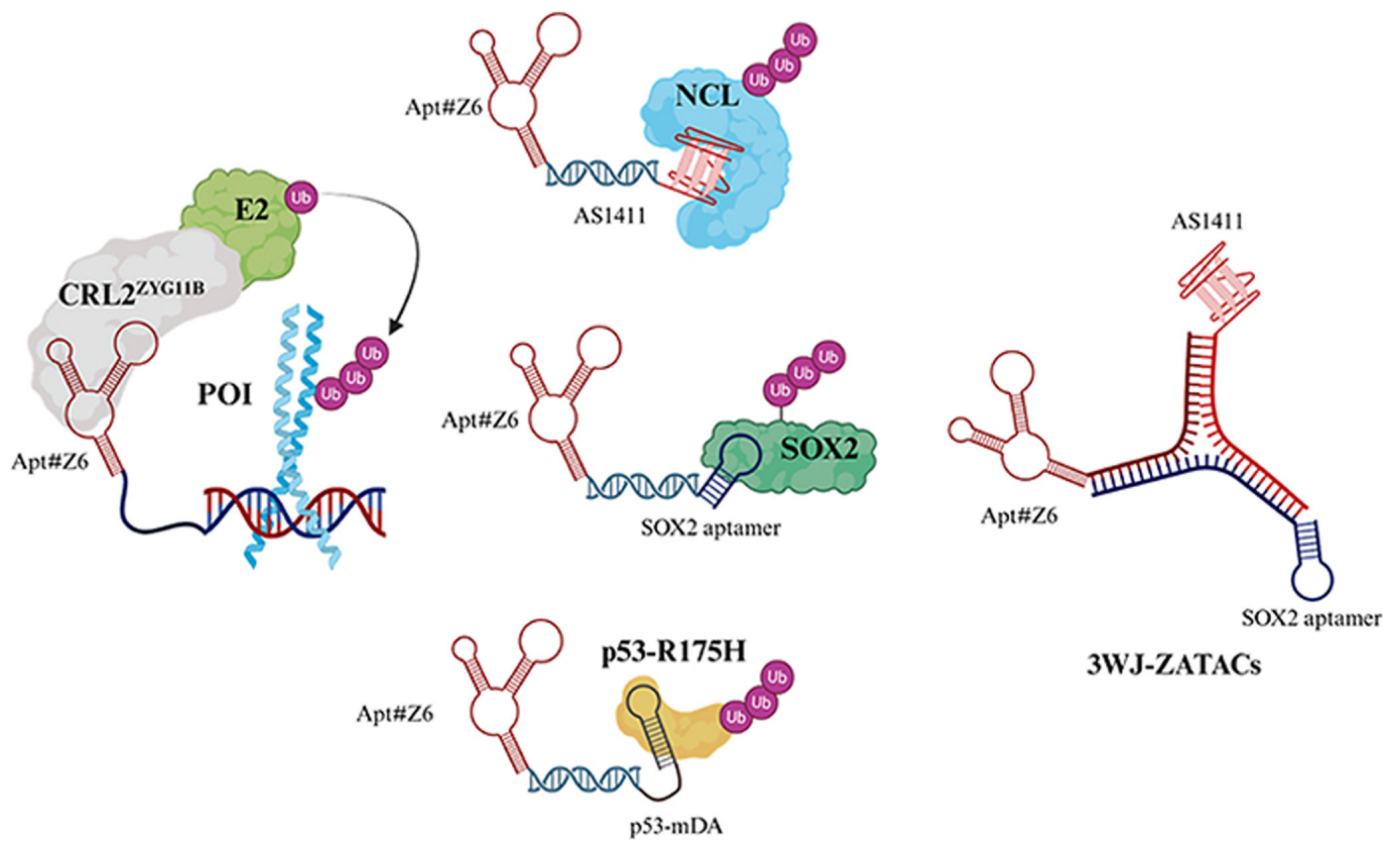
Apt#Z6 binds to CRL2^ZYG11B^, which can be leveraged for the degradation of POI, exemplified by NCL, SOX2 and p53-R175H. Figure created with BioRender.com.

Yang and coworkers described a novel approach to expand the range of E3 ubiquitin ligases available for PROTAC design [106]. They employed magnetic bead-based SELEX to identify highly specific aptamers that interact with Zyg-11 family member B (ZYG11B), a substrate receptor of the Cullin 2-RING E3 ubiquitin ligase (CRL2) complex, enabling its use as a novel E3 ligase in PROTAC development. Through this strategy, the aptamer Apt#Z6 was identified. It binds CRL2ZYG11B without interfering with its ubiquitin ligase activity, as confirmed by isothermal titration calorimetry (ITC), showing a dissociation constant of 4.69 μM. Apt#Z6 was leveraged to develop ZYG11B aptamer-based PROTACs (ZATACs) (Fig. 13), which can be synthesized via bioorthogonal chemistry or self-assembly. To evaluate their utility, Halo-ZATACs were created to degrade HaloTag fusion proteins in cells. These ZATACs induced dose- and time-dependent degradation of targets like GFP-HaloTag through a CRL2^ZYG11B^ -dependent proteasomal pathway, achieving a DC_50_ of 44.15 nM. Furthermore, the versatility of the bispecific ZATAC platform by targeting a range of oncogenic and traditionally “undruggable” proteins, including NCL, SOX2, and the p53-R175H mutant was demonstrated (Fig. 13). To address delivery challenges associated with aptamer-based PROTACs, DNA three-way junction (3WJ)-based ZATACs (3WJ-ZATACs) were developed (Fig. 13). These Y-shaped nucleic acid constructs integrate an additional aptamer that targets proteins overexpressed on tumor cell surfaces—such as NCL—enabling tumor-specific uptake without the need for transfection reagents. *In vivo* studies showed that 3WJ-ZATACs achieved tumor-specific delivery and exhibited potent antitumor activity, supporting their potential for targeted cancer therapy [106].

## 5 Synthetic strategies for oligonucleotide-based PROTACs

**Fig. 14:**
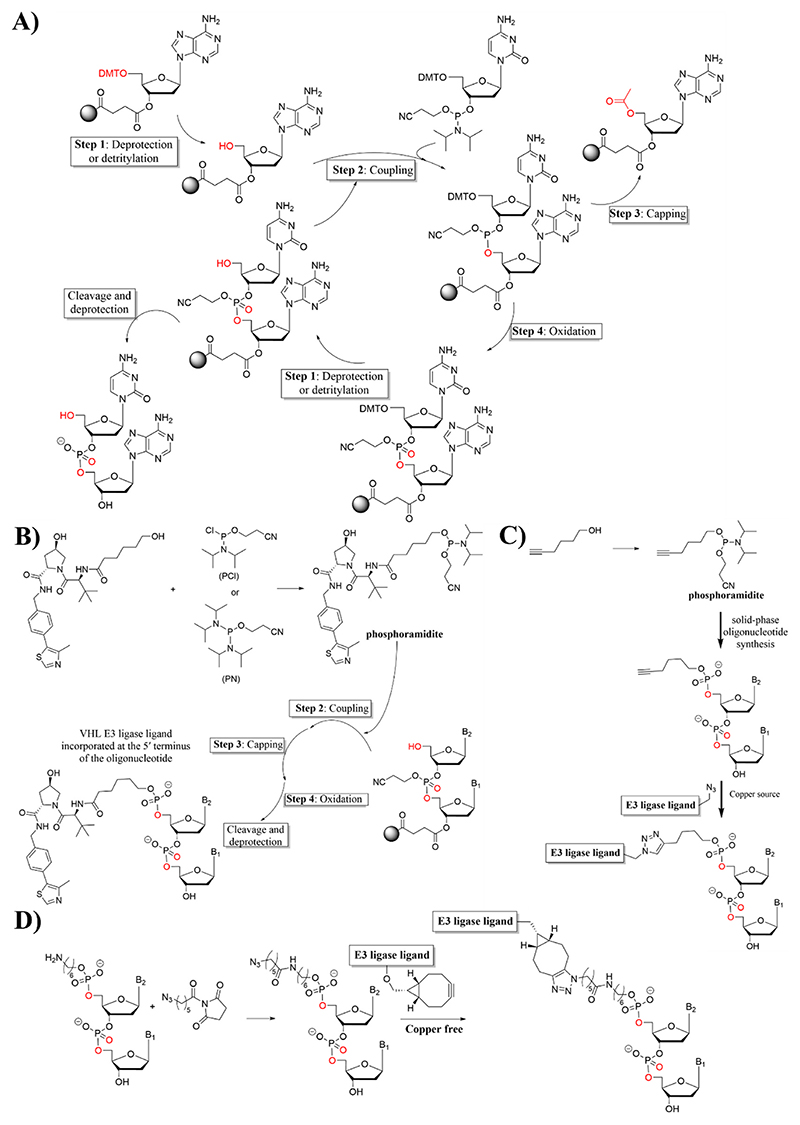
Solid-phase synthesis of modified oligonucleotides and post-synthetic conjugation to an E3 ligase ligand. **A)** General phosphoramidite-based solid-phase oligonucleotide synthesis cycle. **B)** Synthesis of a functionalized phosphoramidite bearing an E3 ligase ligand and its incorporation at the 5′ terminus of an oligonucleotide using standard solid-phase synthesis conditions. **C)** Post-synthetic conjugation strategy using a terminal alkyne-modified oligonucleotide followed by click chemistry with an azide-functionalized E3 ligase ligand. **D)** SPAAC between an azide-modified oligonucleotide and a cyclooctyne-functionalized E3 ligase ligand.

The synthesis of oligonucleotide-based PROTACs involves various methods for attaching an E3 ligase ligand to a nucleic acid-based warhead. Among the most explored synthetic strategies are solid-phase synthesis, click chemistry, and the annealing of modified oligonucleotide strands. The synthesis of the oligonucleotide warhead typically employs standard phosphoramidite solid-phase oligonucleotide synthesis, which follows a four-step cycle: deprotection, coupling, capping, and oxidation (Fig. 14A). In the first step, the detritylation will lead to a free 5′-hydroxyl group, which will react with an activated nucleoside phosphoramidite. After that, any unreacted hydroxyl groups will be capped with an acetyl group. Finally, phosphite triester will be oxidized to the corresponding phosphate. This process is conducted on a solid support, such as controlled pore glass (CPG) or polystyrene beads, using an automated synthesizer [107–109]. Final cleavage from the solid support and global deprotection afford the fully deprotected oligonucleotide. (Fig. 14A). Some oligonucleotide-based PROTACs can also be constructed by annealing two modified DNA strands, each bearing a distinct functional domain (e.g., two AS1411 aptamers [89], or AS1411 linked to V7t1) [90]. These strands may be joined by short poly(A)/poly(T) linkers, allowing for bivalent or bispecific targeting while preserving native secondary structures such as G-quadruplexes.

For oligonucleotide-based PROTACs synthesized via phosphoramidite chemistry, the E3 ligase ligand is first derivatized with a linker and converted into a phosphoramidite (Fig. 14B). This can be achieved using 2-cyanoethyl diisopropylchlorophosphoramidite (PCl) [110] or 2-cyanoethyl *N*,*N*,*N*′,*N*′-tetraisopropylphosphorodiamidite (PN) [111] (Fig. 14B). The modified E3 ligase ligand is then incorporated at the 5′ terminus of the oligonucleotide during automated synthesis. The resulting oligonucleotides can be annealed with complementary strands to form duplexes, as demonstrated in the synthesis of LEF1 and ERG O’PROTACs [25]. It is important to note that direct modification using pomalidomide-derived linkers has shown lower purity and yield, likely due to hydrolysis of the pomalidomide moiety under standard phosphoramidite synthesis conditions [25].

Phosphoramidite chemistry also enables the incorporation of alkyne (Fig. 14C) or azide (Fig. 14D) groups into oligonucleotides, facilitating click chemistry approaches. Among these, Copper-Catalyzed Azide–Alkyne Cycloaddition (CuAAC) (Fig. 14C) is the most widely used due to its bioorthogonality, high efficiency, and compatibility with aqueous conditions [112–115]. CuAAC has been employed in the synthesis of various oligonucleotide-based PROTACs, including oligoTRAFTACs [24], STAT3 targeting PROTACs [37] and ER targeting PROTACs [48, 53,55]. This method supports post-synthetic functionalization and is adaptable to a range of linker lengths and flexibilities.

In addition, Strain-Promoted Azide–Alkyne Cycloaddition (SPAAC) has been utilized for PROTAC design, particularly with dibenzocyclooctyne (DBCO)- and bicyclo[6.1.0]non-4-yne (BCN) (Fig. 14D). The synthesis of TF-PROTACs [30], for instance, involves conjugating azide-modified DNA oligomers (N_3_-ODN) with BCN-modified VHL E3 ligase ligands (VHLL-X-BCN). This strategy allows for straightforward purification using commercial oligonucleotide purification kits, with the resulting TF-PROTACs being ready for cellular transfection after annealing. Regarding linker composition, short alkyl chains (1 to 5 methylene groups) and polyethylene glycol (PEG) linkers yield the best results in terms of reaction efficiency. Furthermore, increasing the amount of VHLL-BCN ligand and extending reaction time enhances SPAAC yields. For example, using a 10-fold excess of VHLL-BCN for 4 h resulted in a click efficiency above 60%, which increased to over 90% after 16 h, highlighting the robustness of this approach [30].

## 6 Outlook

While conventional drug discovery has been successful for proteins with well-defined binding sites, a large portion of the human proteome remains “undruggable” due to the absence of suitable ligand-binding pockets. TPD offers an innovative, event-driven pharmacology able to eliminate disease-causing proteins. Among TPD strategies, PROTACs have emerged as powerful tools capable of selectively degrading proteins of interest through induced ubiquitination and proteasomal degradation. TF, long considered undruggable, represent a particularly promising class of targets for PROTAC development. The incorporation of oligonucleotide decoys into PROTAC design further expands the range of targetable proteins, providing enhanced specificity through sequence-directed recognition. Since early reviews on the topic regarding the use of oligonucleotides in the TPD field [16,21,22], their focus was in the use of these as POI ligands, because of their nature to achieve high potency and selectivity being binding motifs of the POI. Their potential as POI ligand has been explored in the degradation of a range of undruggable targets, such as c-Myc, NF-κB, brachyury and others. Databases such as JASPAR [116] offer an important resource for identifying transcription factor binding motifs, which can guide the rational design of oligonucleotide-based PROTACs targeting specific DNA-binding proteins associated with disease states. However, their role have been expanded, now being used as linkers, to control spacing and orientation between the POI ligand and E3 ligase ligand, which can optimize the ternary complex formation, through the use of DTACs [105]. Nucleotides can be also used to increase the valence of PROTACs, allowing the utilization of different parts, that can incorporate cell-specific delivery of the PROTAC and recognition motifs to interact with different targets [104,106]. In addition, they can be also used as E3 ligase ligand, such as in the case of ZATACs [106]. Table 1 summarizes all the data presented in this review.

Antisense oligonucleotides (ASOs) and small interfering RNAs (siRNAs) have historically been used to either silence gene expression or repress translation of mRNA. For example, conformationally stable ASOs targeting c-Myc lead to a robust reduction in tumor burden and improved overall survival in Myc-overexpressing cancers [117]. Similarly, siRNAs/ASO-based strategies targeting NFκB (RelA/p65), E2F and p53, [118–120] have been shown to inhibit the ability of the malignant cells to proliferate or even resist apoptosis. Therapeutic approaches based on shRNA, siRNA, ASOs, and oligonucleotide decoys have mostly resulted in positive preclinical outcomes, with several candidates either currently in or having completed Phase I/II clinical trials [121]. These aspects, along with antisense oligonucleotide-based drug candidates currently in clinical trials have been reviewed in details elsewhere [122–126]. Despite these advances, major challenges limit the therapeutic application of siRNA- and ASO-based cancer therapeutics, including inefficient targeted delivery to tumor cells, and in particular a major limitation is the off-target activity of these approaches [127] which is distinct from the ligand specific nature of oligonucleotide decoy-based PROTACs.

Oligonucleotides possess potential limitations for clinical application, due to their high molecular weight, anionic nature and chemical instability. Usually for evaluation, to pass through the cell membrane, they require transfection reagents, which are cytotoxic and limits further clinical development. Previously, a positively charged polymer, polyethylenimine, served as the transfection carrier for delivering LEF1 OP-V1 into human cancer cell-xenotransplanted mice [25]. However, polyethylenimine exhibits dose-dependent cytotoxicity [128]. Thus, it is of paramount importance to employ delivery systems that are biodegradable, biocompatible, and non-toxic. Several delivery platforms have been evaluated in the development of oligonucleotide-based drugs, including ASOs, siRNA, and aptamers. Lipid nanoparticles (LNPs) and N-acetylgalactosamine (GalNAc) conjugates have been explored and are clinically validated for targeted delivery of oligonucleotides to the liver [129]. LNPs use lipids to form multicomponent nanoparticles that encapsulate the oligonucleotide cargo [130]. In contrast, GalNAc conjugation does not require formulation, and delivery relies on the recognition of the GalNAc sugar by the asialoglycoprotein receptor, which is expressed on the surface of hepatocytes and mediates internalization of the conjugated oligonucleotide [131–133]. Additional delivery platforms include polymeric nanoparticles [134], cell-penetrating peptides (CPPs) [135], and other ligand-targeted formulations [136]. These strategies aim to improve tissue specificity and expand oligonucleotide delivery beyond hepatic targets toward extrahepatic tissues.

One of the major challenges of oligonucleotide-based drugs is related to their stability, which is due to susceptibility to nucleases. Chemical modifications are employed to enhance stability, prolonging circulation and improving therapeutic efficacy [137]. Incorporating chemical modifications, such as PS and MOE, into the oligonucleotide backbone can enhance stability, increase nuclease resistance, and improve pharmacokinetic properties [137,138]. The use of PS backbone modifications improves another important limitation associated with oligonucleotide-based drugs, namely rapid renal clearance. PS backbone modifications increase protein binding (e.g., to albumin and other blood proteins), thereby slowing renal clearance [139]. However, this modification has also been linked to sequence-unspecific off-target effects [140], as observed in an oligonucleotide-based PROTAC targeting ERα [53]. While most oligonucleotide-based PROTACs developed so far have employed PS and MOE modifications, other chemical modifications are known to improve oligonucleotide properties. For example, phosphorodiamidate morpholino oligomer (PMO) and peptide nucleic acid (PNA) modifications provide a neutral backbone and increased resistance to nucleases [141,142]. Lastly, locked nucleic acids (LNAs) promote conformational restriction of the sugar ring and demonstrate improved nuclease resistance and binding affinity [143].

The solid-phase phosphoramidite oligonucleotide manufacturing process involves synthesis, cleavage and deprotection, purification and isolation, being able to achieve high yields (50%) and purity (90%) [144,145]. In order to generate oligonucleotides for clinical and commercial use, solid-phase synthesis of oligonucleotide is associated with limitations regarding the cost of the resin, large consumption of expensive chemicals and limited scalability (<10 kg batches) [144,146]. In addition, large amounts of organic and aqueous waste are produced during the synthesis and purification. Beyond that, atom economy is extremely poor due to the use of several protecting groups in the process [144]. In order to increase the scale of the oligonucleotide synthesis, new approaches have been undertaken exploring liquid-phase approaches [147]. Oligonucleotide-based PROTACs synthesis employs solid-phase phosphoramidite chemistry, click chemistry, or the annealing of modified oligonucleotide strands. Incorporating azide or alkyne modifications into the oligonucleotides enable post-synthetic conjugation via click chemistry, particularly CuAAC and SPAAC. These methods offer high efficiency, bioorthogonality, and flexibility. In addition, methods with minimal purification steps provide an important platform for quick screening. While these methods show to be suitable for *in vitro* study of oligonucleotide-based PROTACs, additional limitations are related with scalability.

Oligonucleotide-based PROTACs are still at an earlier stage of clinical development than their small-molecule counterparts; however, several features of this modality make it attractive for future therapeutic development. First, oligonucleotide-based PROTACs can target transcription factors and other “undruggable” proteins that are not readily amenable to small-molecule PROTAC strategies. In addition, they leverage sequence-defined recognition elements and chemistries that build on longstanding experience with ASOs and siRNAs [21,148]. However, clinical application of oligonucleotide-PROTACs shows pharmacokinetic and pharmacodynamic challenges relative to small-molecule counterparts. In contrast, small-molecule PROTACs have been administered via oral and parenteral routes and their ADME/PK properties have been extensively investigated and characterized [149, 150]. Oligonucleotide-based PROTACs are expected to possess PK/PD features similar to other therapeutic nucleic acids, such as poor oral absorption, prolonged tissue retention, slow elimination from certain organs (e.g., liver and kidney), and sequence- and chemistry-dependent plasma protein binding [64,151,152]. Finally, for oligonucleotide-based PROTACs, additional complexity needs to be considered, such as the requirement to achieve sufficient intracellular concentrations to support productive ternary complex formation, as well as potential hook effects at higher exposures that may complicate dosing and narrowing the therapeutic window [153–155]. Thus, clinical application of oligonucleotide-based PROTACs will require careful integration of PROTAC PK/PD principles with the learnings from ASO and siRNA modalities.

Ticking all the boxes in PROTAC design, serving as the POI ligand, linker, and E3 ligase ligand, oligonucleotides are promising candidates for the development of novel drugs and tool compounds able to chemically knockdown diverse targets. This is especially true for approaches that allow the synthesis of these compounds with minimal purification steps, as oligonucleotide decoys functionalized for bioorthogonal chemistry are readily available from commercial suppliers.

## Figures and Tables

**Table 1:** Summary of oligonucleotide-based PROTACs.

Target Protein	Lead Compound	Platform	E3 Ligase	Degradation	Primary Cell Lines	In Vivo Data (Y/ n)	Source
Oligonucleotides decoys-based PROTACs.							
NF-κB	HP14	TRAFTAC	VHL (via haloPROTAC, e.g., HP14)	Significant degradation occurred at concentrations over 10 μM	HEK293 stable cells expressing dCas9-HT7	n	23
Brachyury	HP14	TRAFTAC	VHL (via haloPROTAC, e.g., HP14)	Significant degradation occurred at concentrations lower than 10 μM	HEK293 stable cells expressing dCas9-HT7	Y	23
c-Myc	OT7	oligoTRAFTAC	VHL	Significant degradation at 50 nM	HeLa cells	n	24
Brachyury	OT3/OT17	oligoTRAFTAC	VHL	Significant degradation at 50 nM	HEK293T, UM-Chor1	Y	24
LEF1	LEF1 OP-V1	O’PROTAC	VHL	DC_50_ = 25 nM	PC-3, DU145	Y	25
ERG	ERG OP-C-N1	O’PROTAC	crbn	DC_50_ = 182.4 nM	VCaP	n	25
p65 (NF-κB)	dNF-B #15 and dNF-B #16	TF-PROTAC	VHL	Degradation of NF-kB (p65) was observed in HeLa cells when treated with 10 μg/mL	HeLa, HEK293T	n	30
E2F1	dE2F #16/#17	TF-PROTAC	VHL	Degradation was observed in HeLa cells when treated with 10 μg/mL or 25 μg/mL	HeLa, HEK293T	n	30
ETV6/ETV6-fusions	d(GGAA)_3_ #8	TF-PROTAC	VHL	Significant degradation was observed in A673 cells when treated with 10 μg/mL	A673, MHH-ES-1	n	32
STAT3	POM-STAT3	Decoy Oligo-PROTAC	crbn	DC_50_ = 261 nM	NCI-H2087, MCF7	n	37
STAT3	C-STAT3DPROTAC	CpG-Oligo-PROTAC	crbn	85% degradation at 2 μM in A20 cell without transfection reagents	A20, OCI-Ly3	Y	19
ERα	LCL-ER(dec)	Decoy Oligo-PROTAC	IAP	Significant degradation at concentration of 10 μM	MCF-7	n	48
ERα	LCL-ER(dec)-H46	Hairpin Decoy-PROTAC	IAP	Significant degradation at concentration of 10 μM	MCF-7	n	53
ERα	LCL-ER(dec)-allS	PS-modified Decoy-PROTAC	IAP	Significant degradation at concentration of 0.1 μM	MCF-7	n	53
ER	CPP/HDO-PROTAC	CPP/HDO-PROTAC	IAP	Degradation at 10 μM without the need for transfection reagents	MCF-7	n	55
c-Myc	MP-16/MP-17	Decoy Oligo-PROTAC	VHL	Significant degradation at concentration of 0.4 μM	SK-Hep-1, SNU-398	Y	58
LIN28A	ORN3P1	RNA-PROTAC	VHL	Concentration of 2 μM showed an approximate 50% of degradation	NT2/D1	n	60
**Aptamer-based PROTACs**							
Nucleolin (NCL)	ZL216	Aptamer-PROTAC	VHL	DC_50_ = 13.5 nM	MCF-7, BT474	Y	82
Nucleolin (NCL)	dNCL#T1	Inducible Apt-PROTAC	CRBN	Significant degradation at concentration of 0.5 μM	MCF-7	n	83
STAT3	1411-S3-2	ANM-PROTAC	MDM2 (via NCL bridge)	DC_50_ = 136.3 nM	HeLa	Y	88
MDM2	AS1411-VH032	AS1411-VH032 PROTAC	VHL	DC_50_ = 35.82 nM	HeLa, A549	Y	89
MDM2	HomoAS1411	AS1411-AS1411 PROTAC	MDM2 (via NCL bridge)	DC_50_ = 71.30 nM	HeLa, A549	Y	89
VEGF165	AS1411-V7t1	Dual Aptamer-PROTAC	MDM2 (via NCL bridge)	DC_50 _= 109 nM	HeLa, HUVEC	Y	90
c-MET	AS1411-SL1-2	Dual Aptamer-PROTAC	MDM2 (via NCL bridge)	DC_50_ = 199.9 nM	mnng/hos	Y	93
p53-R175H	dp53m-RA	RNA Aptamer-PROTAC	crbn	DC_50_ = 1.06 μM	H1299, SKBR3	n	98
p53-R175H	dp53 m	DNA Aptamer-PROTAC	crbn	Significant degradation at concentration of 0.25 μM	H1299-p53-R175H, Detroit 562	Y	100
c-Myc	ProMyc	Aptamer-PROTAC	crbn	DC_50_ = 5.02 nM	HCT116 cells	Y	102
ZBP1	C-PROTAC	Covalent PROTAC	VHL	DC_50_ = 25.69 nM	A549, HeLa	Y	103
**Oligonucleotides as linkers**							
STAT3	AS-TD2-PRO	DNA Tetrahedron PROTAC	VHL	Degradation of 40% at concentration of 0.25 μM and 90% at 1 μM	MCF-7	Y	104
Cyclin D1-CDK4/6	DTAC-V1	DNA-Templated PROTAC(DTAC)	crbn	CDK6 DC_50_ = 55 nM CDK4 DC_50_ = 12 nM Cyclin D1 DC_50_ = 20 nM	U-251, MM1.S, AU565, A549	Y	105
**Oligonucleotides as E3 ligases**							
Nucleolin (NCL)	NCL-ZATAC#20	ZYG11B Aptamer-PROTAC (ZATAC)	ZYG11B	DC_50_ = 158 nM	MCF-7, A549	Y	106

## Data Availability

Data will be made available on request.
